# Variability of Prokaryotic Community Structure in a Drinking Water Reservoir (Marathonas, Greece)

**DOI:** 10.1264/jsme2.ME11253

**Published:** 2011-10-05

**Authors:** Despoina S. Lymperopoulou, Konstantinos Ar. Kormas, Amalia D. Karagouni

**Affiliations:** 1 National and Kapodistrian University of Athens, Faculty of Biology, Department of Botany, Microbiology Group, 157 81 Athens, Greece; 2 Department of Ichthyology & Aquatic Environment, School of Agricultural Sciences, University of Thessaly, 384 46 Volos, Greece

**Keywords:** *Archaea*, *Bacteria*, 16S rRNA, diversity, freshwater reservoir

## Abstract

The structure of the *Bacteria* and *Archaea* community in a large drinking water reservoir (Marathonas, Greece; MR) was investigated in October 2007 and September 2008, using 16S rRNA gene clone libraries. The bacterial communities were more diverse than archaeal communities (Shannon diversity index *H*′ 0.81–3.28 and 1.36–1.77, respectively). The overall bacterial community composition was comparable to bacterioplankton community described in other freshwater habitats. Within the *Bacteria*, *Betaproteobacteria* dominated, while representatives of *Alpha-*, *Gamma-* and *Deltaproteobacteria* also occurred. Other important phyla were *Actinobacteria* and *Bacteroidetes*, while representatives of *Acidobacteria*, *Cyanobacteria*, *Chloroflexi*, *Planctomycetes* and *Verrucomicrobia* were also retrieved. Several phylotypes in *Alpha-* and *Betaproteobacteria* and *Bacteroidetes* were related to bacteria capable of cyanotoxin degradation and with aromatic compounds/iron oxidizers or polymer degraders. *Euryarchaeota* dominated (60.5%) the *Archaea* community mostly with phylotypes related to *Methanobacteriales* and *Methanosarcinales*. Among the *Thaumarchaeota*, the two most abundant phylotypes were affiliated (97% similarity) with the only cultivated mesophilic thaumarchaeote of marine origin, *Nitrosopumilus maritimus*. Temporal and spatial comparison of the prokaryotic community structure revealed that three of the most abundant prokaryotic phylotypes, belonging to *Actinobacteria*, were recovered from all sites both years, suggesting that these *Actinobacteria* could be important key players in MR ecosystem functioning.

In freshwater habitats, bacterioplankton mediates crucial biogeochemical processes. It plays an important role in nutrient cycling (10) through the breakdown of organic matter and the remineralization of nutrients (70), while at the same time it controls water quality and the fate of pollutants (11); however, our understanding of bacterioplankton dynamics in freshwater is limited and the factors that drive the actual composition of bacterial communities (34) are not currently known. One of the primary questions remains whether planktonic prokaryotes are characterized by as yet unpredictable dynamics in community structure or recurrent patterns of their community composition (67). Although the variability of freshwater prokaryotic community composition on both spatial and temporal scales has been demonstrated in several studies (*e.g.* 10, 67, 68), drinking water reservoirs remain one of the most understudied systems in this field.

The most common and informative approach for studying the composition of microbial communities in aquatic environments involves the construction of 16S rRNA gene libraries, since aquatic prokaryotes are difficult to obtain in pure culture (24, 25). This approach has revealed previously unsuspected prokaryotic diversity and has led to the accumulation of a vast number of sequences in global databases, affiliated to phylogenetic groups that were either unknown or thought to be absent from the aquatic ecosystems (25). In total, 21 phyla have been recovered from lake epilimnia, while five (*Proteobacteria*, *Actinobacteria*, *Bacteroidetes*, *Cyanobacteria* and *Verrucomicrobia*) are the most commonly recovered (40). Furthermore, recent studies have identified many uncultured and apparently ubiquitously distributed bacterial phylotypes in freshwater lakes and reservoirs (13, 39, 72), but still little is known about the temporal variation of bacterial taxa in such systems. Most of these groups do not include cultured representatives and it is rather difficult to assign putative roles in the absence of a strain whose physiology has been characterized. The assignment of ecophysiological roles to environmental 16S rRNA gene sequences demands complementary culture derived knowledge, especially in the case of *Archaea* inhabiting mesophilic environments (36), whose ecology and functional role remain poorly characterized compared to *Bacteria*(43). Three phyla of *Bacteria*, *Proteobacteria*, *Actinobacteria*, and *Bacteroidetes*, usually occur at high frequencies in clone libraries from many lakes (2, 10, 13, 17, 72), while the presence of mesophilic *Archaea* in the planktic prokaryotic community has been sporadically reported in different lakes such as Lake Cadagno (7), Mono Lake (21) and Lake Pavin (32).

The diversity-function relation is not always obvious (35) and it is strongly associated with the metacommunity effect and the functional redundancy of the ecosystem (5, 35). On the other hand, among the few links that have been established between environmental conditions and the ecology of aquatic bacterial groups is that different phyla are related to the autochthonous or the allochthonous origin of carbon (22). Allochthonous carbon sources tend to be more influential in lakes with short hydraulic retention time (57) and terrestrial bacteria play a crucial role in shaping the community structure (33).

Reservoirs differ considerably from natural lakes in respect of basic physical, chemical and biological processes, which might result in different prokaryotic dynamics than in natural lakes. Maybe the most prominent effect is caused by the reservoir’s hydrological regime (58). Due to operational management, their water volume can vary considerably over short, and often unpredicted, time scales, so reservoirs experience shorter retention times than lakes, intense water level fluctuations, as well as periodic pulses of mixing depending on their use (62). Such changes have been found to have profound effects among other biota on the phytoplankton community of a reservoir (23, 38) and their direct or indirect effect on prokaryotic communities remains unknown. In this paper, we aimed at depicting the differences between the *Bacteria* and *Archaea* community structure based on 16S rRNA gene diversity at the end of the warm period in a drinking water reservoir (Marathonas) in the city of Athens, Greece, in two consecutive years.

## Materials and Methods

Marathonas Reservoir (MR) is located in Attiki, Greece, about 35 km northeastern of the city of Athens. It covers 2.5 km^2^ with mean depth of 15 m. Its drainage basin is 118 km^2^ with a total water circulation of 14,400,000 m^3^ per year, when the yearly mean rainfall value is 580 mm. MR was the main water resource for Athens from its establishment in 1931 until 1959. Currently, MR’s water content is adequate only for a few days to supply Athens and its use is occasionally supplementary. Water retention time is about 187–200 days (37). During sampling, surface water temperature in the centre of the MR was 18.4°C and 22.3°C on 30 October 2007 and 8 September 2008, respectively.

**Fig. 1:**
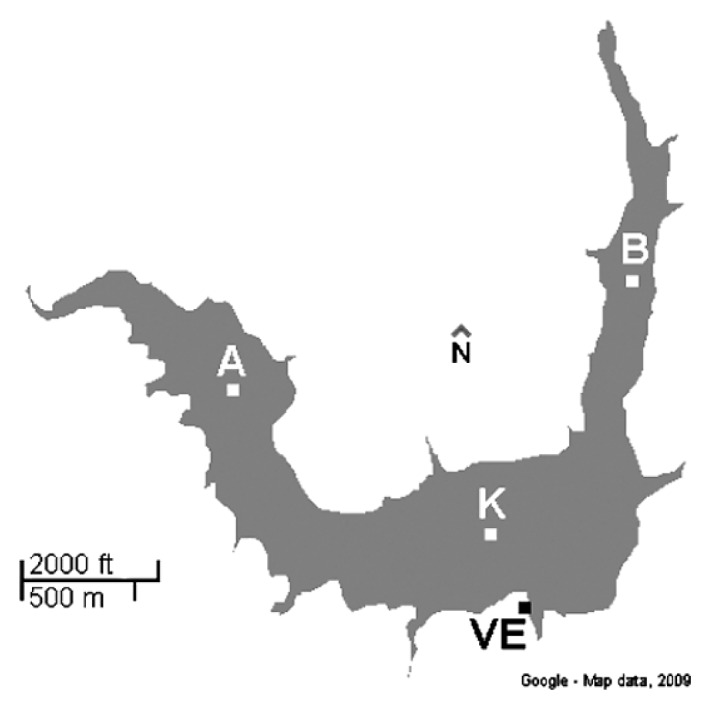
Sampling sites at the Marathonas Reservoir (MR), Greece.

Water samples were collected from four different sites of the reservoir (Fig. 1): A (38°10′32.59″N–23°53′1.33″E), B (38°10′ 45.41″N–23°54′18.77″E), K (38°10′9.91″N–23°53′58.91″E) and VE (water collection tower, 38°9′55.77″N–23°54′0.68″ E). Samples were collected from approximately 0.5 m below the surface in sterile plastic carboys. The samples were kept cool and in darkness until they were processed (<4 hours after collection). Water samples of 12 L from each site were filtered through 0.22 μm isopore polycarbonate filters (Millipore, Molsheim, France) and filters were stored at −20°C until further processing.

DNA was extracted using the Ultra Clean Mega Soil DNA Isolation Kit (MoBio Laboratories, Carlsbad, USA) following the manufacturer’s protocol and dissolved in 1 mL PCR water. The DNA was diluted 1:10 with PCR water before PCR amplification to overcome persistent PCR inhibition problems. *Bacteria* 16S rRNA genes were amplified using the primers BAC-8F (5′-AGAGT TTGATCCTGGCTCAG-3′) (31) and BAC-1390R (5′-TGTACA CACCGCCCGTC-3′) (28). An initial denaturation step at 94°C for 1 min was followed by 28–30 PCR cycles (94°C denaturation for 45 s; primer annealing at 52.5°C for 45 s; and primer extension at 72°C for 2 min), and a final 7 min elongation step at 72°C. The *Archaea* 16S rRNA genes were amplified using nested PCR. For the first amplification round, the primers ARC-8F (5′-TC CGGTTGATCCTGCC-3′) (61) and ARC-1390R (5′-GACGGGCG GTGTGTGCAA-3′) (28) were used. These PCR products were re-amplified in a second PCR run using the primers ARC-344F (5′-ACGGGGYGCAGCAGGCGCGA-3′) (46) and ARC-915R (5′-GTGCTCCCCCGCCAATTCCT-3′) (56). Each PCR run consisted of a 1 min pre-PCR hold at 94°C, followed by 25–35 cycles consisting of a 45 s denaturation step at 94°C, a 45 s annealing step at 52.5°C, a 2 min elongation step at 72°C. A final 7-min elongation step at 72°C was added. The number of cycles was determined for each sample after cycle optimization. PCRs were repeated with different cycle numbers and the lowest number of cycles that yielded a product was then used for cloning and sequencing in order to avoid differential representation of 16S rRNA genes with low and high copy numbers.

PCR products were visualized on a 1.2% agarose gel under UV light, bands were excised, and PCR products were extracted with the Nucleospin Extract II PCR Clean-up kit (Macherey-Nagel GmbH, Düren, Germany) following the manufacturer’s protocol. The PCR products were cloned using the TOPO XL PCR cloning kit (Invitrogen, Carlsbad, USA) and chemically competent cells, according to the manufacturer’s specifications. For each clone library, randomly selected clones containing the insert of the appropriate length (*ca.* 1,400 bp and 550 bp for *Bacteria* and *Archaea*, respectively) were grown in liquid LB medium with kanamycin and their plasmids were purified using the Nucleospin Plasmid QuickPure kit (Macherey-Nagel GmbH, Düren, Germany) for DNA sequencing.

Sequence data were obtained by capillary electrophoresis (Macrogen, Tokyo, Korea) using the BigDye Terminator kit (Applied Biosystems, Carlsbad, USA) with the primers M13F (-20) and M13R. Each sequence read was approximately 850 bp, and for each individual bacterial clone, forward and reverse reads were assembled. The length of the bacterial 16S rRNA gene sequences after assembly was *ca.* 1,390 bp. The sequences were screened for chimeras using the PINTAIL program (http://www.bioinformatics-toolkit.org/Web-Pintail/). All putative chimerical sequences were excluded from further analysis.

To detect the closest relatives, all sequences were compared with the BLAST function (http://www.ncbi.nlm.nih.gov/BLAST/). Sequence data were aligned using the ClustalW aligning utility (http://www.ebi.ac.uk/Tools/clustalw2/index.html) and phylotypes or operational taxonomic units were defined as sequences showing ≥98% homology to each other. All unique phylotypes were then compiled, along with sequences obtained from GenBank (www.ncbi.nlm.nih.gov) and phylogenetic trees were constructed using the neighbor-joining method implemented in the MEGA4 software (60). Bootstrapping was performed with 1,000 replicates to assign confidence levels to the tree topology.

Library clone coverage was calculated by the formula [1−(*n*1*/N*)] (14), where *n*1 is the number of operational taxonomic units (OTU) represented by only one clone and *N* is the total number of the clones examined in each library. Diversity estimations were based on the Shannon-Wiener index *H*′ (54) and the Pielou evenness index *J*(42, 54). Microbial community similarities were determined using the Morisita index of similarity (66). The Morisita indices of similarity were further analyzed by cluster analysis using the PAST software (18).

### Nucleotide sequence accession numbers

Sequences of unique phylotypes found in this study have GenBank accession numbers GQ340065–GQ340365 for *Bacteria* and GQ340366–GQ340402 for *Archaea*.

## Results and Discussion

In this study, we investigated the 16S rRNA gene diversity of *Archaea* and *Bacteria* at three surface (*ca.* 0.5 m) sites and the water collection tower (VE) of the Marathonas drinking water reservoir (MR) at the end of the warm period in two consecutive years (September 2007 and October 2008). To the best of our knowledge, this is the first reported concomitant study of bacterial and archaeal community structures in a drinking water reservoir. Incorporating the prokarytic component into a reservoir’s known biota can provide valuable information such as the occurrence of potentially toxic cyanobacteria (37) or other microbes of public health interest (*e.g.* 29). In the current study, however, no such prokaryotes were identified, suggesting that MR water does not pose a direct risk to public health.

The satisfactory coverage of our clone libraries (Fig. S1A and B), along with the high number of singleton phylotypes, suggested that the majority of the existing species richness in the studied samples has been revealed. This renders the use of diversity indices feasible and the Shannon–Wiener diversity index has been suggested as the most appropriate for estimating the diversity of prokaryotic communities (20).

### Archaea

The detection of archaeal 16S rRNA gene clones was feasible only after nested amplification of all sampling sites, implying a rather low *Archaea* abundance in the MR. Similar observations have been previously attributed either to the *Archaea*’s poor adaptation to the prevalent conditions or to their low growth rates, which do not favor their dominance in lake ecosystems (36).

A total of 105 archaeal 16S rRNA gene sequences from all sampling sites, 53 in 2007 (A: 11, B: 9, K: 16, VE: 17) and 52 in 2008 (A: 17, B: 11, K: 10, VE: 14) were retrieved and attributed to 15 and 22 phylotypes, respectively (Table S1). According to the Good’s C estimator for library clone coverage (25), curves from all sampling sites in both 2007 and 2008 reached a plateau above 0.80 (Fig. S1A). The majority of the sequences (59.5%) belonged to the *Euryarchaeota*, while the remaining phylotypes were identified either as *Crenarchaeota* (8.1%) or as *Thaumarchaeota* (32.4%) (Fig. S2).

The dominance of *Euryarchaeota* over *Thaumarchaeota* and *Crenarchaeota* in both sampling periods agrees with the concept that planktonic communities of these three groups may occupy different ecological niches in the same ecosystem (9, 19). Around 50% of the found *Euryarchaeota* phylotypes belonged to *Methanobacteriales* and 32% to *Methanosarcinales* (Fig. S2), which are commonly recovered archaeal groups in freshwater and marine sediments, respectively (4, 6). The presence of such methanogens at the MR surface could rather be attributed to the perturbation of sediment during water removal at the reservoir, as they occur in freshwater lakes and river sediments (36, 47) mediating methane production in the final step of the anaerobic degradation of organic matter (4, 43).

The three *Crenarchaeota* phylotypes that were recovered, although not related to any cultivated representatives, were affiliated with microorganisms from rice roots or soil and were only retrieved in 2008 from sites A and K.

Among the phylotypes in the *Thaumarhaeota*, only two were moderately related (90–96%) to a cultivated representative of the phylum, Candidatus ‘*Nitrososphaera gargensis*’. Seven phylotypes remained unclassified, while five sequences were related (90–97%) to *Nitrosopumilus maritimus*. Among these five sequences were the two most abundant *Thaumarchaeota* phylotypes in 2007 from sites A and K. *N. maritimus* is the only cultivated mesophilic thaumarchaeota of marine origin (27) and has never been isolated from a freshwater environment. *N. maritimus* is a common chemoautotroph in oligotrophic, devoid of organic matter environments and lives by oxidizing ammonia to nitrate (27).

**Fig. 2:**
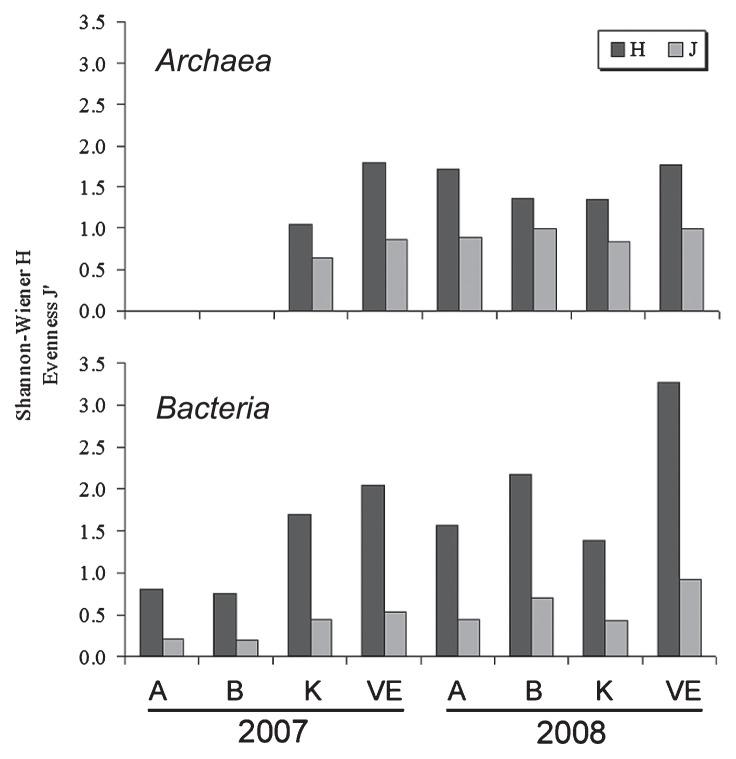
Shannon-Wiener diversity index of the prokaryotic communities in Marathonas Reservoir (MR), Greece.

In 2007, the *Archaea* diversity index *H*′ (Fig. 2) ranged between 0 (Sites A and B with only one phylotype) and 1.18 (Site VE), while in 2008 it ranged from 1.36 (Site K) to 1.77 (Site VE). The variation of the evenness index (*J*′=0–0.99) at each site indicated similarly unequal phylotype distribution.

**Fig. 3:**
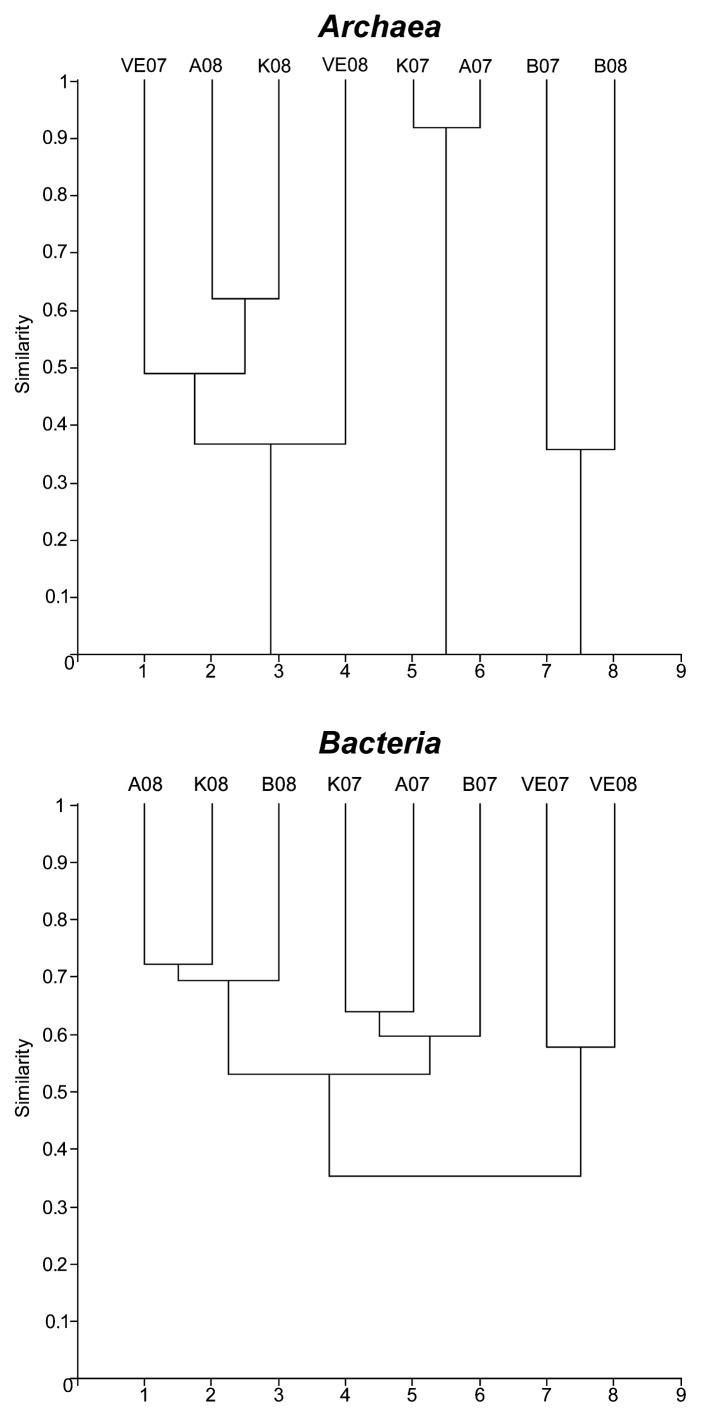
Cluster analysis of the prokaryotic communities in Marathonas Reservoir (MR), Greece.

Cluster analysis (Fig. 3) suggested that the *Archaea* communities were highly similar (>90%) only at sites A and K in 2007 but unrelated to any other station. All other stations were clustered together with similarities ranging from 35% to 65%. No common phylotype was found at all four sampling sites in both years. In particular, libraries A07 and K07 were highly similar (>90%) forming a very distinct cluster from all other stations, due to the presence of the most abundant phylotypes A07-01-ARC and K07-01-ARC (both sequences were clustered within the *Nitrosopumilales*). In contrast, sites B and VE in 2007 were not related to each other at all. Site B07 formed a distinct cluster with site B08 due to the retrieval of phylotype B07-01 in site B in both sampling years, but these sites shared less than 40% similarity due to the retrieval of three more phylotypes in site B in 2008. In 2008, sites A, K and VE formed a cluster with similarity values among them ranging from *ca.* 35% to 65%, while site VE07 was distantly clustered in the same group. The differential residence time of the water in the collection tower (VE) between the two years, as a result of the variable water supply needs, could have imposed different selection of the prevailing *Archaea*.

### Bacteria

A total of 342 and 261 bacterial 16S rRNA gene clones were retrieved in 2007 (A: 88, B: 83, K: 79, VE: 92) and 2008 (A: 74, B: 57, K: 59, VE: 71) from all four sampling sites, and these were attributed to 182 and 118 phylotypes, respectively (Table S2). According to Good’s C estimator for the libraries clone coverage (25), curves from all sampling sites in both 2007 and 2008 reached a plateau above 0.70 (Fig. S1B).

**Fig. 4:**
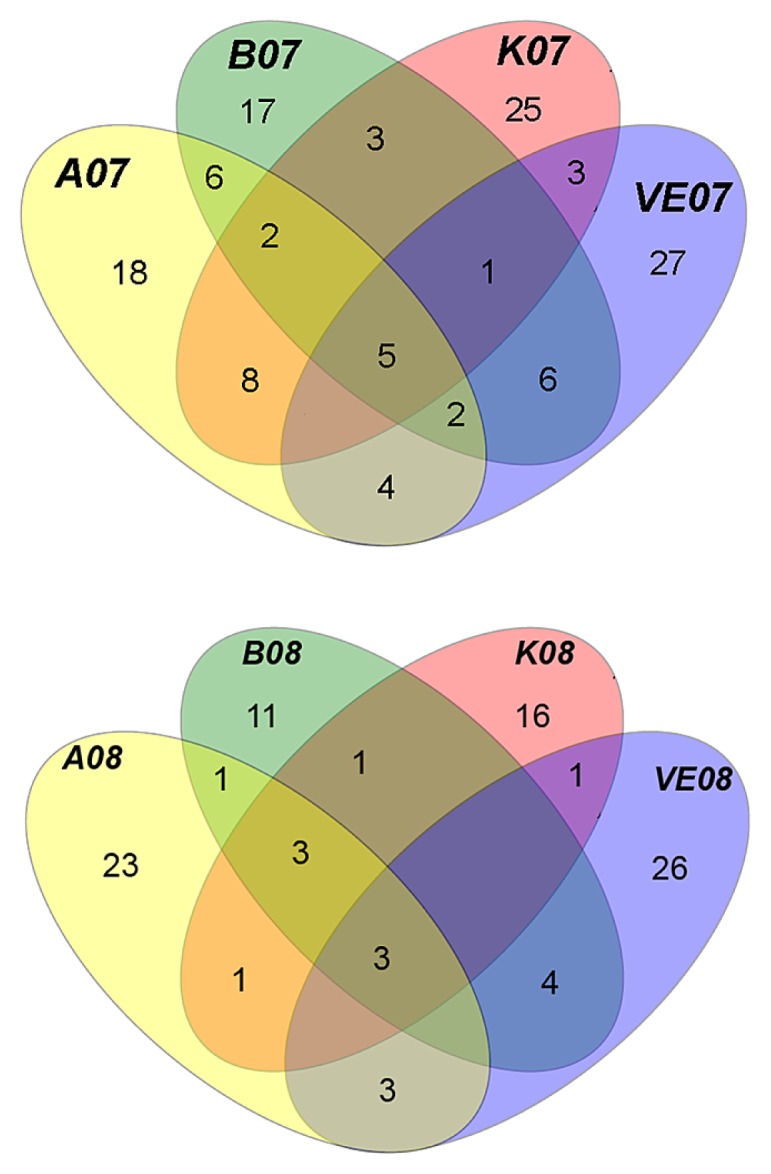
Venn diagrams of the bacterial phylotypes found in Marathonas Reservoir (MR), Greece, in 2007 (top) and 2008 (bottom).

Only three phylotypes were recovered from all sites in both years and belonged to *Actinobacteria* (Fig. 4). The majority of the phylotypes (39%) belonged to the *Proteobacteria* (Fig. S3, 4, 5 and 6) in 2007, while in 2008 *Actinobacteria* (Fig. S7) dominated (33.9%). Five classes of *Proteobacteria* were represented, but not all were retrieved from all sites; *Deltaproteobacteria* were retrieved only from station B08. *Bacteroidetes* (Fig. S8) also comprised a considerable fraction of the reservoir’s species richness (15.4% and 21.2% in 2007 and 2008, respectively). The remaining phylotypes were affiliated to six known phyla (*Acidobacteria*, *Chloroflexi*, *Cyanobacteria*, *Firmicutes*, *Planctomycetes*, *Verrucomicrobia*), while 2.7% in 2007 and 4.2% in 2008 remained unaffiliated (Fig. S9 and 10).

*Proteobacteria* dominated (36% of all *Bacteria*) at the MR surface. In particular, *Betaproteobacteria* prevailed (44.4% of the *Proteobacteria*), followed by *Alphaproteobacteria* (38.9%), while *Gamma-*, *Delta-* and *Epsilonproteobacteria* phylotypes also occurred. *Betaproteobacteria* dominate in freshwater bacterial communities (8, 64) and may reach high abundance in lakes of diverse trophic status (12, 69). Although four to six phylogenetic clusters of freshwater *Betaproteobacteria* have been proposed (13, 72), the β-I (*Rhodoferax*/GKS16) and β-II (*Polynucleobacter necessarius*) clades (55, 67, 68) attract most scientific interest, while much less is known about the cosmopolitan β-III and β-IV clades (13). In our study, we were able to distinguish *Betaproteobacteria* at the family level. In lower taxa, the majority of the phylotypes belonged to *Burkholderiales* and the related phylotypes were distributed in the families of *Comamonadaceae*, *Alcaligenaceae*, *Burkholderiaceae* and *Oxalobacteraceae*. Within the *Burkholderiaceae*, two singletons were retrieved from sites A and B in 2007 that were closely related to the *Polynucleobacter* sp. cluster (15, 72), which is exclusively known in freshwater systems with diverse and sometimes contrasting climatic and ecological features (15, 33, 55, 68). The majority of non-*Burkholderiales* phylotypes was related to the methylotrophic order of *Methylophilales*. Within the family of *Comamonadaceae*, some representative sequences were affiliated with previously described β-I sequences, such as PIB-18 from Piburger Sea (52), while other sequences of this family were directly related to *Paucibacter toxinovorans*, which degrades cyanobacterial hepatotoxins (microcystins and nodularin) (45). Members of the family *Sphingomonadaceae* within the *Alphaproteobacteria* are also known to degrade cyanobacterial toxins (3). In our study, phylotypes clustered within the *Sphingomonadaceae* were retrieved from almost all clone libraries (apart from B and K in 2008), indicating that degradation of microcystins might occur in the lake since MR in known to host potentially toxic *Cyanobacteria*(37).

*Actinobacteria* was the second most abundant phylum (24% of the retrieved phylotypes). *Actinobacteria* are typically soil bacteria and before the application of cultivation-independent techniques it was believed that their presence in freshwater systems was allochthonous (17); however, it is well documented that *Actinobacteria* comprise a large fraction (10–60%) of bacterioplankton in diverse freshwater habitats with different features, including lakes and rivers (2, 10, 13, 17, 65). Indigenous freshwater *Actinobacteria* lineages have been identified (65, 72) and belong mainly to the acI–acIV clusters (2, 13, 65, 70). In our study, 60% of the retrieved phylotypes were not related to any cultured representatives of *Actinobacteria* but some were related to certain phylotypes retrieved from Wisconsin (39) and Adirondack lakes (41). These phylotypes seem to belong to the acI cluster and it is speculated (41) that they are practically identical to sequences retrieved mostly from freshwater lakes by Warnecke *et al.*(65). Our phylotypes belonged to the order of *Actinomycetales* and within the subcluster acI-A. Within this order, along with phylotypes related to the genera *Nocardia*, *Cryobacterium*, *Cellulomonas* and *Blastococcus*, three sequences were affiliated to the acI-B sub-cluster (41). The above findings support the cosmopolitan character of cluster acI, which has mainly been attributed to their ability to avoid protistan predators because of their small size (39, 65).

*Bacteroidetes* was the third most abundant group (17.7%) in the MR. These microorganisms are commonly recognized as hydrolytic fermentative degraders of polymers in mainly anaerobic habitats, including freshwater sediments (26, 50). Phylotypes belonging to the *Flavobacteriales* and *Sphingobacteriales* co-occurred, though it is believed that salinity serves as a selective force which restrains *Flavobacteria* mainly in marine habitats (5); however, *Flavobacteria* are not absent from freshwater (10, 11, 13, 72). Twenty-eight phylotypes were grouped within the *Cytophagales*, including sequences that were practically identical to *Cytophaga* sp., *Hymenobacter* sp. and *Flectobacillus lacus. Cytophaga*-like bacteria have been found in Lake Constance, the Mindelsee and the Meldsee (SW Germany) (48). Both *Cytophagales* and *Flavobacteriales* have been associated with harmful algal blooms (49) based on their algicidal activity and antagonism towards other bacteria (51). Within the order of *Sphingobacteriales*, one doubleton was affiliated with Candidatus ‘*Aquirestis calciphila*’. This is a cosmopolitan species, detected in 62% of the studied freshwater samples, found in hard water lakes and isolated from regions of temperate, tropical and subtropical zones (16).

Less abundant phylotypes were grouped with the *Acidobacteria*, *Firmicutes*, *Cyanobacteria*, *Planctomycetes*, *Verrucomicrobia* and *Chloroflexi* or remained unaffiliated. The retrieval of *Acidobacteria* from freshwater reservoirs is rare and the majority of this phylum’s representatives are of terrestrial origin, while its ecophysiological role is uncertain (44). *Firmicutes* comprise a small fraction of the bacterial community in some freshwater systems that have been detected (10, 57) and in our study only singletons were retrieved. All of the *Cyanobacteria* phylotypes found in the MR belonged to the order *Chroococcales*(37). *Chloroflexi* exhibit low recovery rates in freshwater habitats (22). The four *Chloroflexi*-related phylotypes found were distantly related to *Dehalococcoides ethenogenes* and Candidatus ‘*Chlorothrix halofila’. Planctomycetes* can be found in a variety of habitats but their relative abundance in most cases is low (10). Most of the *Planctomycetes* phylotypes in the reservoir were related to sequences from Lake Manzala, Lake Fayateville or other aquatic habitats, while five others formed a monophyletic group distantly related to a clone from Crater Lake (CL500-3) (63). Within the *Verrucomicrobia*, a group of three phylotypes was retrieved and was related to a phylotype from the Adirondack lakes (41). Typical freshwater *Verrucomicrobia*(73) seem to be widespread in Scandinavian lakes (10, 73), but have also been retrieved from the Greek lakes of Kastoria and Doirani during cyanobacterial water blooms (30) and from other eutrophic lakes (70, 71), suggesting a strong association with cyanobacterial blooms. Among the unaffiliated phylotypes, one monophyletic group was related to clone CL500-48. This clone belongs to the phylum of *Armatimonadetes* (formerly known as OP10 candidate division) (59), a group of mostly uncultivated bacteria that were initially isolated and characterized from Crater Lake (63), but its presence is ubiquitous in freshwater habitats (72).

In 2007 sampling, the *Bacteria* diversity index *H*′ (Fig. 2) ranged between 0.81 (Site A) and 2.04 (Site VE), while in 2008 sampling it ranged from 1.38 (Site K) to 3.28 (Site VE). The distribution of phylotypes in each site’s populations varied significantly (*J*=0.2–0.92), indicating similarly unequal phylotype distribution.

Cluster analysis of *Bacteria* (Fig. 3) showed that all sites within the lake were grouped together for each year, while the communities from the two water collection tower samples (VE) formed a distinct and more distant group. In 2007 stations A, B and K were slightly similar (>60%), but VE was not similar to these three stations. In 2008 stations A, B and K were more similar (>70%) than in 2007, but site VE did not exhibit any similarity to them. Phylotype A07-14, related to *Rhodobacter* sp., plays an important role in this clustering, since it was retrieved from all sampling sites in both years apart from VE in 2008. *Rhodobacter* has been associated with active denitrification in the presence of sulfide-free flow water in the Kama River (53). The distant clustering of the reservoir sites between the two years is attributed to A07-43, an actinobacterial phylotype which was retrieved from all sites in 2007, but was practically absent in 2008. The water collection tower (VE) communities were more similar to each other than to any other in the lake sampling sites, since they formed a distinct cluster exhibiting about 60% similarity between 2007 and 2008.

*Bacteria* diversity was higher than *Archaea* diversity for all sites, which is generally the case for clone libraries constructed from the same sampling location (1). In highly unstable systems, such as MR due to rainfall, land run-off, water withdrawal and flushing (23), the stability of the system could be due to the presence of different organisms which are able to perform the same metabolic function, allowing the mineralization of organic matter, regardless of the shifts observed in microbial populations (1). Such phylotypes may exhibit ecophysiological overlap, at least for functions considered crucial for survival in the reservoir’s environment (functional redundancy) (35).

In conclusion, we revealed bacterial and archaeal 16S rRNA gene diversity in the Marathonas drinking water reservoir. Only three phylotypes were recovered from all sites in both years and belonged to *Actinobacteria*, but were not related to any cultivated representatives. These phylotypes were also the most abundant sequences, along with a phylotype clustered with *Rhodobacterales*. The differences between the archaeal and bacterial communities consisted of lower diversity of *Archaea* and different community similarity between stations. These results indicate that the planktonic communities of *Archaea* and *Bacteria* exhibit different temporal and spatial patterns in a drinking water reservoir.

## Supplementary Material
