# Supplementary material for: Variability of Prokaryotic Community Structure in a Drinking Water Reservoir (Marathonas, Greece)
Source: Microbes Environ. 2011 Oct 5;27(1):1–8. doi: 10.1264/jsme2.ME11253 (PMC4036031; doi:10.1264/jsme2.ME11253)
Supplement: Supplementary file 1 [file 27_1_s1.pdf]

SUPPLEMENTARY MATERIAL

Lymperopoulou *et al*., submitted to "Microbes and Environments"

Table S1. Occurrence of archaeal phylotypes in three water column stations (A, B, K) and the water collection tower of Marathonas Reservoir, Greece, on 30 October 2007 and 08 September 2008.

	Clone	*No. of similar (≥98%) clones*	*Putative affiliation*	*Closest sequence (similarity) [GenBank accession No.]*	*Description*	*Closest organism (similarity) [GenBank accession No.]*
**2007**						
1	A07-01-ARC	11	*Thaumarchaeota*	Clone BI15J2 (99.64 %) [EU490315]	Lake Bourget – France	
2	B07-01-ARC	9	*Euryarchaeota*	Clone s39t24arch30 (99.82 %) [FM957645]	Rice field soil	
3	K07-01-ARC	11	*Thaumarchaeota*	Clone BI15J2 (99.64 %) [EU490315]	Lake Bourget – France	
4	K07-05-ARC	1	*Euryarchaeota*	Clone Hua4-s77 (98.90 %) [EU481582]	Saline wetland	
5	K07-06-ARC	2	*Thaumarchaeota*	Clone HTA-G6 (99.27 %) [AF418938]	Freshwater reservoir	
6	K07-07-ARC	1	*Thaumarchaeota*	Clone SLG-332 (98.55 %) [EU365284]	Soil	
7	K07-10-ARC	1	*Euryarchaeota*	Clone sl3182 (99.82 %) [EU244212]	River Leine sediment	
8	VE07-01-ARC	5	*Thaumarchaeota*	Clone ARC_10SAF2-28 (97.60 %) [DQ782347]	NASA spacecraft assembly facility	
9	VE07-03-ARC	1	*Euryarchaeota*	Clone MH1492_4E (99.46 %) [EU155950]	Rich minerotrophic fen	
10	VE07-04-ARC	5	*Euryarchaeota*	Clone NA5AR52 (99.46 %) [FM957993]	Rice paddy soil	*Methanosarcina* sp. 48 (99.28 %) [EF112192]
11	VE07-05-ARC	1	*Thaumarchaeota*	Clone LR-305 (99.45 %) [DQ302464]	Lonar soda lake (India)	
12	VE07-06-ARC	1	*Euryarchaeota*	Clone QEED1BH111 (93.31 %) [CU916385]	Wastewater sludge	*Methanospirillum hungatei* (89.80%) [AB517987]
13	VE07-07-ARC	2	*Thaumarchaeota*	Clone BuhD-Ar111 (98.91 %) [FM897353]	Uranium mill tailings	Candidatus *Nitrososphaera gargensis* clone RHGA92E13c (96.02%) [EU281335]
14	VE07-09-ARC	1	*Thaumarchaeota*	Clone sscpArc_V5A5 (99.64 %) [FM179191]	Groundwater	
15	VE07-10-ARC	1	*Euryarchaeota*	Clone LL_Koral_24 (98.55 %) [AM503239]	Roots of rice	*Methanospirillum hungatei* (95.83 %) [AY196683]
**2008**						
16	A08-22-ARC	5	*Euryarchaeota*	Clone NA5AR52 (99.46 %) [FM957993]	Soil	*Methanosarcina* sp. 48 (99.28 %) [EF112192]
17	A08-25-ARC	5	*Euryarchaeota*	Clone G19 (99.46 %) [EU910626]	Lake Huron (USA)-sediment	
18	A08-26-ARC	2	*Crenarchaeota*	Clone MH1492_B2C (98.91 %) [EU155996]	Rich minerotrophic fen	
19	A08-27-ARC	1	*Thaumarchaeota*	Clone NG-W-080829_2-8 (99.28 %) [AB550807]	River water	

20	A08-30-ARC	2	*Euryarchaeota*	Clone MD2902-A47 (94.43 %) [EU385840]	Subseafloor sediments	
21	A08-31-ARC	1	*Crenarchaeota*	Clone LL_ADT_46 (98.73 %) [AM503301]	Rice roots	
22	A08-32-ARC	1	*Euryarchaeota*	Clone NG-W-080829_2-3 (96.28 %) [AB550818]	Rice roots	
23	B08-21-ARC	2	*Euryarchaeota*	Clone N4-E2 (96.74 %) [FN691626]	Lake	*Methanobacterium uliginosum* NBRC 105232 (79.93 %) [AB542914]
24	B08-27-ARC	3	*Euryarchaeota*	Clone N4-E2 (93.50 %) [FN691626]	Lake	*Methanobacterium uliginosum* NBRC 105232 (76.23 %) [AB542914]
25	B08-33-ARC	3	*Thaumarchaeota*	Clone ZZ-50 (94.11 %) [HM051131]	Soil	
26	B08-34-ARC	3	*Euryarchaeota*	Clone s39t24arch30 (99.82 %) [FM957645]	Rice field soil	
27	K08-26-ARC	1	*Crenarchaeota*	Clone 2PZ28.1 (98.92 %) [FJ957963]	Wetland	
28	K08-30-ARC	5	*Euryarchaeota*	Clone NA5AR52 (99.46 %) [FM957993]	Soil	*Methanosarcina* sp. 48 (99.28 %) [EF112192]
29	K08-34-ARC	1	*Thaumarchaeota*	Clone CaR3s.19 (93.71 %) [EF014505]	Lake	
30	K08-38-ARC	2	*Thaumarchaeota*	Clone NG-W-080829_2-8 (99.09 %) [AB550807]		
31	K08-39-ARC	1	*Euryarchaeota*	Clone IU-FSC-4_Ar-A011 (98.20 %) [AB380041]	Soil	
32	VE08-23-ARC	2	*Euryarchaeota*	Clone NO-24 (98.38 %) [GQ906627]	River	
33	VE08-25-ARC	3	*Euryarchaeota*	Clone RF_ADT_40 (99.09 %) [AM503211]	Riverbank soil	
34	VE08-26-ARC	2	*Euryarchaeota*	Clone YBSAr06 (99.27 %) [FM165684]	Rice paddy soil	Candidatus *Methanoregula boonei* SN19 (97.64 %) [EU887826]
35	VE08-30-ARC	2	*Euryarchaeota*	Clone 20c-52 (94.55 %) [AJ299201]	Aegean sea sediments	
36	VE08-36-ARC	3	*Euryarchaeota*	Clone rDu915-G2 (99.28 %) [FJ822564]	Acidic bog	*Methanosarcina* sp. HC-2 (98.92 %) [AB288264]
37	VE08-40-ARC	2	*Euryarchaeota*	Clone Mat-ARC-19 (98.26 %) [EU255755]	Freshwater	

Table S2. Occurrence of bacterial phylotypes in three water column stations (A, B, K) and the water collection tower of Marathonas Reservoir, Greece, on 30 October 2007 and 08 September 2008.

	Clone	*No. of similar (≥98%) clones*	*Putative affiliation*	*Closest sequence (similarity) [GenBank accession No.]*	*Description*	*Closest organism (similarity) [GenBank accession No.]*
**2007**						
38	A07-02	1	*Betaproteobacteria*	Clone DP3.2.7 (99. 57 %) [FJ612136]	Dongping Lake water (China)	*Polynucleobacter cosmopolitanus* KF027 (99.64 %) [AB269813]
39	A07-06	1	*Bacteroidetes*	*Sphingobacterium* sp. clone ZS-4-66 (96.94 %) [FN668098]	Lake Zurich	*Sediminibacterium* sp. TEGAF015 (92.23 %) [AB470450]
40	A07-09	2	*Unaffiliated*	Clone YHY15 (98.83 %) [GU305800]	YangHe reservoir/MinYun reservoir	
41	A07-12	1	*Alphaproteobacteria*	Clone D44 35 (99.78 %) [EU580491]	Lake Contance	*Sphingomonas* sp. B18 (99.63 %) [AF410927]
42	A07-13	1	*Unaffiliated*	Crater Lake Clone CL500-48 (99.49 %) [AF316757]	Oligotrophic Crater Lake water	
43	A07-14	6	*Alphaproteobacteria*	Clone DP10.4.4 (99.70 %) [FJ612398]	Lake water	
44	A07-16	1	*Bacteroidetes*	Clone Foos8B_57 (94.89 %) [EU431801]	Calcium carbonate muds at percolating waters	*Tuber borchii* symbiont b-17BO (89.86 %) [AF070444]
45	A07-21	1	*Actinobacteria*	Actinobacterium clone TR1E4 (99.71 %) [EU117953]	Freshwater	*Candidatus Planktophila limnetica* MWH-EgelM2-3.acI (97.82 %) [FJ428831]
46	A07-22	1	*Alphaproteobacteria*	Clone tls2-10 (99.08 %) [DQ463198]	Acid Mine Drainage	*Novosphingobium pentaromativorans* 16-21 (95.19 %) [EU167952]
47	A07-23	3	*Bacteroidetes*	Bacteroidetes clone PRD18D09 (99.56 %) [AY948029]	Temperate River	
48	A07-25	1	*Actinobacteria*	Clone MYS16 (98.50 %) [GU305737]	YangHe reservoir/MinYun reservoir	

49	A07-32	7	*Actinobacteria*	Clone MYS14 (99.85 %) [GU305708]	YangHe reservoir/MinYun reservoir	
50	A07-33	1	*Betaproteobacteria*	Clone ML-5-90.2 (99.86 %) [DQ520167]	Shallow hypertrophic freshwater lake (China)	*Variovorax* sp. EMB 263 (97.89 %) [DQ413166]
51	A07-39	1	*Betaproteobacteria*	Betaproteobacterium clone ADK-CSe02-53 (99.93 %) [EF520468]	Acid Impacted Lake	
52	A07-40	1	*Verrucomicrobia*	Clone SINO895 (97.18 %) [HM129970]	Tibetan Lake (China)	*Luteolibacter algae* A5J-40 (96,92 %) [AB331894]
53	A07-41	1	*Verrucomicrobia*	Clone MYW29 (98.65 %) [GU305773]	YangHe reservoir/MinYun reservoir	*Methylacidiphilum infernorum* V4 CP000975 (83.68 %) [CP000975]
54	A07-42	2	*Acidobacteria*	Clone UG7 (97.87 %) [DQ298003]	H/C contaminated soil	*Solibacter usitatus* Ellin6076 (93.53 %) [CP000473]
55	A07-43	2	*Actinobacteria*	Actinobacterium clone TW1C9 (99.85 %) [EU117978]	Freshwater	
56	A07-44	1	*Acidobacteria*	Clone Elev_16S_1240 (98.44 %) [EF019878]	Rhizosphere	*Solibacter usitatus* Ellin6076 (92.20 %) [CP000473]
57	A07-46	1	*Betaproteobacteria*	Clone 3C003438 (99.64 %) [EU802045]	Chesapeak Bay, MD (USA)	*Comamonadaceae* bacterium BP-1b (97.17 %) [AY145570]
58	A07-54	1	*Chloroflexi*	Clone 35-7 (99.34 %) [DQ833481]	Sediment	
59	A07-55	1	*Bacteroidetes*	Clone YHW4 (97.92 %) [GU305829]		
60	A07-57	1	*Actinobacteria*	*Nocardia* sp. TFS 668 (98,98 %) [EF216351]	Sediment	
61	A07-60	1	*Bacteroidetes*	Clone B21.11 (99.85 %) [GU559811]	Anaerobic fermentation of *Microcystis* blooms	
62	A07-62	4	*Cyanobacteria*	*Microcystis aeruginosa* 0BB35S02 (99.48 %) [AJ635430]	Basin of Bubano, Imola, Italy	
63	A07-63	1	*Bacteroidetes*	Clone PW25.9B (99.71 %) [EU573114]	Oil reservoir	*Flavobacterium* sp. WB3.4-82

						(99.85 %) [AM934665]
64	A07-64	4	*Bacteroidetes*	*Sphingobacteriales* clone SF68 (99.35 %) [AJ697704]	Mesotrophic Lake	*Chimaereicella* sp. A8-7 (97.28 %) [EU313811]
65	A07-65	4	*Actinobacteria*	Clone MYW3 (99.85 %) [GU305754]	YangHe reservoir/MinYun reservoir	
66	A07-66	1	*Betaproteobacteria*	Clone 117ds10 (95.70 %) [AY212568]	Equine Feacal Contamination	*Aquamonas mexican* AQ11 (94.27 %) [AB120965]
67	A07-68	1	*Alphaproteobacteria*	Clone 5C230894 (98.66 %) [EU803336]	Lake Gatun, Panama	*Roseomonas* sp. SK 65 (96.21 %) [AM991117]
68	A07-70	2	*Bacteroidetes*	Clone nbw992e03c1 (98.74 %) [GQ045991]	Human Skin Microbiome	*Tuber borchii* symbiont b-17BO (95.11 %) [AF070444]
69	A07-73	4	*Gammaproteobacteria*	Clone MC1_16S_55 (98.99 %) [EU662624]	Sulfidic water (Movile Cave, Romania)	*Rheinheimera chironomi* K19414 (97.70 %) [DQ298025]
70	A07-76	2	*Verrucomicrobia*	Clone MYW38 (99.57 %) [GU305779]	MiYu reservoir water	*Lentimonas marisflavi* IMCC2112 (84.98 %) [EF157839]
71	A07-77	2	*Alphaproteobacteria*	Alphaproteobacterium clone ZS-2-22 (99.55 %) [FN668055]	Lake Zurich	*Rhodobacter* sp. Jip03 (97.17 %) [AB122032]
72	A07-78	4	*Betaproteobacteria*	*Curvibacter* putative symbiont of *Hydra magnipapillata* genomic scaffold HmaUn_WGA71069_1 (99.71 %) [FN543107]	Symbiont of *Hydra magnipapillata*	*Aquamonas mexican* AQ11 (98.42 %) [AB120965]
73	A07-79	3	*Actinobacteria*	Clone MYS13 (100.00 %) [GU305736]	MiYu reservoir water	
74	A07-82	1	*Alphaproteobacteria*	Alphaproteobacterium HIBAF003 (99.92 %) [AB452982]	Freshwater Bacterioplankton	*Brevundimonas* sp. DR12 (99.24 %) [FJ447545]
75	A07-84	1	*Alphaproteobacteria*	Clone A9_10.4_2 (99.39 %) [FJ716899]	Marine sediment from Cullercoats, Northumberland, UK	*Agrobacterium* sp. P128 (99.39 %) [EU195949]
76	A07-87	1	*Actinobacteria*	Clone SINI1012 (96.56 %) [HM126680]	Tibetan Lake (China)	*Actinomycetales* bacterium GSoil 1621 (90.08 %) [AB245400]
77	A07-88	1	*Bacteroidetes*	Clone 2C229017 (99.35 %) [EU800805]	Delaware Bay, NJ (USA)	*Fluviicola taffensis* RW262 (92.88 %) [AF493694]
78	A07-90	1	*Bacteroidetes*	Clone CV24 (95.92 %) [DQ499283]	Cave system biofilm	*Tuber borchii* symbiont b-17BO

						(90.03 %) [AF070444]
79	A07-91	2	*Bacteroidetes*	Clone FFCH3806<br>(97.92 %) [EU133760]	Soil	*Fluviicola taffensis* RW262<br>(94.23 %) [AF493694]
80	A07-92	2	*Bacteroidetes*	Clone SING780<br>(99.42 %) [HM129373]	Tibetan Lake (China)	
81	A07-95	3	*Betaproteobacteria*	Clone DP10.3.1<br>(99.71 %) [FJ612363]	Dongping Lake<br>Ecosystems (China)	*Curvibacter gracilis*<br>(97.78 %) [AB109889]
82	A07-97	4	*Bacteroidetes*	*Flexibacter* sp.<br>clone ZS-1-345<br>(99.71 %) [FN668151]	Lake Zurich	
83	B07-03	2	*Verrucomicrobia*	Verrucomicrobia clone QEEB2AB02<br>(90.61 %) [CU917669]	Sludge	
84	B07-05	1	*Actinobacteria*	Actinobacterium clone ADK-Moh02-65<br>(96.24 %) [EF520362]	Acid impacted lake	*Cellulomonas* sp. F11<br>(90.98 %) [EU697083]
85	B07-10	1	*Alphaproteobacteria*	Clone 3C002503<br>(99.63 %) [EU801252]	Chesapeake Bay, MD<br>(USA)	Rhizobiales bacterium<br>Mfc52<br>(94.48 %) [AB365487]
86	B07-19	1	*Actinobacteria*	Clone YHW22<br>(99.64 %) [GU305846]	YangHe reservoir/MinYun<br>reservoir (China)	
87	B07-22	1	*Bacteroidetes*	Clone K2-30-6<br>(95.14 %) [AY344418]	Hawaiian archipelago	
88	B07-23	4	*Verrucomicrobia*	Clone MYW38<br>(99.86 %) [GU305779]	YangHe reservoir/MinYun<br>reservoir	*Lentimonas marisflavi*<br>IMCC2112<br>(84.98 %) [EF157839]
89	B07-24	2	*Bacteroidetes*	Clone DP10.2.20<br>(99.64 %) [FJ612348]	Dongping Lake<br>Ecosystems (China)	*Arcicella* sp. MG83<br>(93.21 %) [AJ746140]
90	B07-26	1	*Actinobacteria*	*Nocardia* sp. TFS 359<br>(99.06 %) [EF216366]	Sediment (Trondheim<br>fjord)	
91	B07-29	2	*Actinobacteria*	Clone 2C229335<br>(99.27 %) [EU801038]	Delaware Bay, NJ, USA	
92	B07-30	4	*Bacteroidetes*	Clone DP10.4.3<br>(99.71 %) [FJ612397]	Dongping Lake<br>Ecosystems	
93	B07-31	1	*Gammaproteobacteria*	*Rheinheimera taxanensis* TSWCW<br>(98.41 %) [GQ284452]	Ghats (India)	
94	B07-32	1	*Betaproteobacteria*	Clone 2C229239<br>(99.93 %) [EU800970]	Delaware Bay, NJ, USA	
95	B07-33	4	*Alphaproteobacteria*	Clone DP10.4.4<br>(99.85 %) [FJ612398]	Dongping Lake<br>Ecosystems	

96	B07-34	1	*Betaproteobacteria*	Clone DP10.4.32 (99.64 %) [FJ612414]	Dongping Lake Ecosystems	
97	B07-37	2	*Betaproteobacteria*	Clone 3C003438 (99.42 %) [EU802045]	Chesapeake Bay, MD, USA	*Comamonadaceae* bacterium BP-1b (97.17 %) [AY145570]
98	B07-38	2	*Actinobacteria*	Actinobacterium clone LA1C1 (99.85 %) [EU117788]	Freshwater	*Candidatus Planktophila limnetica* MWH-EgelM2-3.acI (97.96 %) [FJ428831]
99	B07-39	1	*Cyanobacteria*	Clone DP7.1.5 (98.13 %) [FJ612231]	Dongping Lake Ecosystems	*Coscinodiscus radiatus* - chloroplast p148 (88.41 %) [AJ536462]
100	B07-41	2	*Cyanobacteria*	*Limnococcus limneticus* Svet06 (99.77%)[GQ375048]	Eutrophic freshwater fishpond	*Gloeocapsa* sp. PCC 73106 (94.56 %) [AF132784]
101	B07-44	1	*Actinobacteria*	Actinobacterium clone Eub62A911 (98.16 %) [GQ390232]	Lake Pavin	*Sporichthya polymorpha* (91.99 %) [X72377]
102	B07-46	2	*Actinobacteria*	Clone 3C002646 (99.85 %) [EU801375]	Chesapeak Bay, MD, USA	
103	B07-49	1	*Alphaproteobacteria*	Clone D44 35 (99.63 %) [EU580491]	Lake Constance	*Sphingomonas* sp. B18 (99.63 %) [AF410927]
104	B07-50	1	*Chloroflexi*	Clone HTH4 (98.72 %) [AF418964]	Freshwater Reservoir	*Kouleothrix aurantiaca* MYSI-A (86.28 %) [AB079639]
105	B07-52	3	*Betaproteobacteria*	Clone 3C003467 (99.21 %) [EU802072]	Chesapeake Bay, MD, USA	*Rhodoferax* sp. Asd M2A1 (98.28 %) [FM955857]
106	B07-53	1	*Gammaproteobacteria*	*Pseudomonas* sp. CJ11064 (99.50 %) [AF500211]		
107	B07-57	7	*Actinobacteria*	Clone MYW3 (99.71 %) [GU305754]	YangHe reservoir/MinYun reservoir	
108	B07-59	3	*Bacteroidetes*	Clone 2C229017 (99.35 %) [EU800805]	Delaware Bay, NJ, USA	*Fluviicola taffensis* RW262 (92.88 %) [AF493694]
109	B07- 61	4	*Actinobacteria*	Clone MYS21 (99.93 %) [GU305741]	YangHe reservoir/MinYun reservoir	
110	B07-63	1	*Planctomycetes*	Clone DP10.2.53 (94.45 %) [FJ612357]	Dongping Lake Ecosystems	*Planctomycete* GMD16E07 (82%) [AY162118]
111	B07-64	1	*Chloroflexi*	Clone MYS10	YangHe reservoir/MinYun	

				(99.78 %) [GU305734]	reservoir	
112	B07-65	1	*Betaproteobacteria*	Clone DP3.2.7 (99.57 %) [FJ612136]	Dongping Lake Ecosystems	*Polynucleobacter cosmopolitanus* KF027 (99.64 %) [AB269813]
113	B07-66	1	*Alphaproteobacteria*	Alphaproteobacterium clone ZS-2-22 (99.62 %) [FN668055]	Lake Zurich	*Rhodobacter* sp. Jip03 (97.25 %) [AB122032]
114	B07-68	10	*Actinobacteria*	Clone MYY14 (99.85 %) [GU305708]	YangHe reservoir/MinYun reservoir	
115	B07-69	1	*Bacteroidetes*	Clone 3C003104 (98.99 %) [EU801740]	Chesapeake Bay, MD, USA	*Flavobacterium* sp. INBF006 (98.47 %) [AB426577]
116	B07-70	1	*Actinobacteria*	Clone ML-7-114.2 (96.33 %) [DQ520182]	Shallow hypertrophic lake	
117	B07-71	2	*Verrucomicrobia*	Clone NMG8 (91.19 %) [GU183621]	Aquifer	
118	B07-82	1	*Alphaproteobacteria*	*Afipia broomeae* F186 (99.93 %) [U87759]		
119	B07-85	1	*Bacteroidetes*	*Flexibacter* sp. clone ZS-4-376 (95.43 %) [FN668199]	Lake Zurich	*Fluviicola taffensis* RW262 (93.37 %) [AF493694]
120	B07-88	2	*Cyanobacteria*	Clone DP3.2.22 (99.63 %) [FJ612143]	Dongping Lake Ecosystems	*Thalassiosira pseudonana* - chloroplast (99.03 %) [EF067921]
121	B07-89	1	*Planctomycetes*	Clone K0029 (97.11 %) [FJ820433]	Wuliangsuhai Lake (China)	
122	B07-90	2	*Alphaproteobacteria*	Clone A9_10.4_2 (99.17 %) [FJ716899]	Marine sediment from Cullercoats, Northumberland, UK	*Agrobacterium* sp. P128 (99.16 %) [EU195949]
123	B07-93	1	*Acidobacteria*	Acidobacterium clone ZS-4-79 (99.57 %) [FN668206]	Lake Zurich	*H. foetida* TMBS4-T (DSM 6591-T) (91.44 %) [X77215]
124	B07-94	1	*Bacteroidetes*	Clone MYY7 (95.51 %) [GU305702]	YangHe reservoir/MinYun reservoir	
125	K07-02	1	*Alphaproteobacteria*	Rhodospirillaceae bacterium Dia-1 (99.08 %) [EU678309]	Lake Constance	*Azospirillum* sp. Mat2-1a (91.45 %) [AY118222]
126	K07-03	2	*Actinobacteria*	Actinobacterium clone ME7A10 (99.78 %) [EU117760]	Lake epilimnion	
127	K07-04	2	*Betaproteobacteria*	Betaproteobacterium clone MI1G10	Temperate river	*Comamonadaceae*

				(99.93 %) [FJ916099]		bacterium BP-1b (97.46 %) [AY145570]
128	K07-05	1	*Bacteroidetes*	*Fluviicola* sp. clone XZELH61 (99.86 %) [EU703159]	Lake bacterioplankton	*Fluviicola taffensis* RW262 (91.08 %) [AF493694]
129	K07-06	1	*Verrucomicrobia*	Clone MYW29 (99.43 %) [GU305773]	YangHe reservoir/MinYun reservoir	*Methylacidiphilum fumariolicum* SolV (84.49 %) [EF591088]
130	K07-07	1	*Betaproteobacteria*	Betaproteobacterium clone QEEB2DE10 (97.42 %) [CU917998]	Sludge	*Derxia gummosa* IAM13946 (95.12 %) [AB089482]
131	K07-10	1	*Cyanobacteria*	*Microcystis aeruginosa* 0BB35S02 (99.63 %) [AJ635430]	Basin of Bubano, Imola, Italy	
132	K07-11	1	*Actinobacteria*	Actinobacterium clone LT1B7 (99.85 %) [EU117819]	Freshwater	
133	K07-13	1	*Cyanobacteria*	Clone DP7.1.5 (94.67 %) [FJ612231]	Dingpong Lake Ecosystems	
134	K07-14	1	*Cyanobacteria*	Clone DP3.2.22 (97.56 %) [FJ612143]	Dongping Lake Ecosystems	*Thalassiosira pseudonana* - chloroplast (96.97 %) [EF067921]
135	K07-18	1	*Actinobacteria*	Clone SINN551 (96.92 %) [HM128624]	Tibetan Lake (China)	*Candidatus Planktoluna difficilis* MWH-7Well8 (95.60 %) [AM939566]
136	K07-20	1	*Alphaproteobacteria*	Clone DP10.4.4 (98.07%) [FJ612398]	Dongping Lake Ecosystems	
137	K07-21	1	*Betaproteobacteria*	Clone Hot Creek 29 (98.56 %) [AY168738]	Biofilm	*Leptothrix* sp. S1.1 (98.49 %) [DQ241397]
138	K07-22	2	*Unaffiliated*	Crater Lake clone CL500-48 (99.41 %) [AF316757]	Oligotrophic Crater Lake water	
139	K07-23	5	*Actinobacteria*	Clone MYS21 (99.93%) [GU305741]	YangHe reservoir/MinYun reservoir	
140	K07-26	3	*Actinobacteria*	Actinobacterium clone ZS-2-02 (99.64 %) [FN668289]	Lake Zurich	
141	K07-34	1	*Bacteroidetes*	Sphingobacteriales Clone SF68 (99.28 %) [AJ697704]	Mesotrophic lake	*Algoriphagus aquatilis* A8-7 (97.21 %) [EU313811]
142	K07-35	1	*Cyanobacteria*	Clone DP3.2.22 (99.26 %) [FJ612143]	Dongping Lake Ecosystems	*Thalassiosira pseudonana* - chloroplast (98%) [EF067921]
143	K07-36	1	*Planctomycetes*	Clone DP10.2.53	Dongping Lake	*Planctomycete* GMD16E07

				(94.23 %) [FJ612357]	Ecosystems	(82%) [AY162118]
144	K07-38	1	*Betaproteobacteria*	Clone 2C228142 (99.71 %) [EU800149]	Delaware Bay, NJ, USA	*Bordetella avium* 197N (96.83 %) [AM167904]
145	K07-40	1	*Alphaproteobacteria*	Clone M3B31 (98.56 %) [FJ439862]	Estrogen-degrading membrane bioreactors	*Hirschia baltica* (90.87 %) [AJ421782]
146	K07-42	1	*Alphaproteobacteria*	*Sphingomonas* sp. A1-8 (99.93 %) [AY512600]	Industrial site	
147	K07-45	1	*Planctomycetes*	Clone FGL12_B49 (99.71 %) [FJ437884]	Fayetteville Green Lake water-NY	*Isophaera* sp. (Schlesner 657) (88.93 %) [X81960]
148	K07-46	1	*Alphaproteobacteria*	Alphaproteobacterium clone D2H08 (98.37 %) [EU753667]	Freshwater stromatolites (Ruidera Pools National Park-Spain)	*Erythrobacter* sp. JL993 (96.15 %) [DQ985049]
149	K07-48	1	*Betaproteobacteria*	Clone 42ds5 (97.77 %) [AY212737]	Fecal contamination	*Pelomonas saccharophila* H-Z9 (96.47 %) [AB495144]
150	K07-49	1	*Firmicutes*	*Bacillus* sp. SGE2 (92.90 %) [AY556409]		
151	K07-52	1	*Gammaproteobacteria*	Clone DP3.2.13 (98.92 %) [FJ612139]	Dongping Lake Ecosystems	*Rheinheimera* sp. HMD2012 (98.49%) [GQ374921]
152	K07-54	3	*Betaproteobacteria*	*Curvibacter* putative symbiont of *Hydra magnipapillata* genomic scaffold HmaUn_WGA71069_1 (99.64 %) [FN543107]	Symbiont of *Hydra magnipapillata*	*Aquamonas fontana* AQ11 (98.28 %) [AB120965]
153	K07-56	1	*Actinobacteria*	Actinobacterium clone TR1A10 (99.93 %) [EU117930]	Lake epilimnion	
154	K07-63	2	*Betaproteobacteria*	Clone b91 (98.13 %) [EU919798]	Bacterioplankton	*Aquabacterium* sp. P-136 (97.57 %) [AM412127]
155	K07-64	1	*Betaproteobacteria*	Clone DP10.4.32 (99.29 %) [FJ612414]	Dongping Lake Ecosystems	
156	K07-73	1	*Betaproteobacteria*	Betaproteobacterium clone 10-90-ArvAB (99.13%) [AB425059]	Alvaneu / Arvadi spring	*Hydrogenophaga* sp. EMB 75 (98/68 %) [DQ413154]
157	K07-75	1	*Bacteroidetes*	Sphingobacteria clone ADK-SGh02-93	Acid impacted lake	*Tuber borchii* symbiont

				(92.17 %) [EF520603]		b-1BO (87.88 %) [AF070444]
158	K07-79	7	*Alphaproteobacteria*	Clone DP10.4.4 (99.78 %) [FJ612398]	Dongping Lake Ecosystems	
159	K07-81	2	*Alphaproteobacteria*	Clone D44 35 (99.70%) [EU580491]	Lake Constance	*Sphingomonas* sp. B18 (99.85 %) [AF410927]
160	K07-82	2	*Betaproteobacteria*	Betaproteobacterium clone ZS-2-04 (99.57 %) [FN668029]	Lake Zurich	*Curvibacter gracilis* (97.49 %) [AB109889]
161	K07-84	1	*Actinobacteria*	Clone SINN551 (99.27 %) [HM128624]	Tibetan Lake (China)	*Candidatus Planktoluna difficilis* MWH-7Well8 (97.83 %) [AM939566]
162	K07-85	1	*Actinobacteria*	Actinobacterium clone LA1C3 (99.56 %) [FJ916093]	Temperate lakes	*Actinomycetales* bacterium GP-5 (90.49 %) [AY145533]
163	K07-87	2	*Actinobacteria*	Actinobacterium clone TW1C9 (99,85 %) [EU117978]	Freshwater	
164	K07-90	1	*Gammaproteobacteria*	*Pseudomonas* sp. CJ11064 (99.43 %) [AF500211]		
165	K07-91	1	*Actinobacteria*	Clone 2C228795 (99.85 %) [EU800635]	Delaware Bay, NJ, USA	*Candidatus Planktophila limnetica* MWH-EgelM2-3.acI (97.96 %) [FJ428831]
166	K07-93	6	*Betaproteobacteria*	RB363 clone (98.56 %) [AB240354]	Rhizospere	*Paucibacter* sp. Asd M1A1 Asd M1A1 (98.48 %) [FM955854]
167	K07-94	1	*Actinobacteria*	Actinobacterium clone CB11A12 (99.20 %) [EF471696]	Estuarine bacterioplankton	
168	K07-96	1	*Bacteroidetes*	*Flectobacillus lacus* CL-GP79 (98.42 %) [DQ112352]	Hypertrophic pond	
169	K07-98	7	*Actinobacteria*	Actinobacterium clone ZS-4-333 (99.49 %) [FN668241]	Lake Zurich	
170	K07-99	1	*Acidobacteria*	UG7 clone (97.87 %) [DQ298003]	H/C contaminated soil	*Solibacter usitatus* Ellin6076 (93.53 %) [CP000473]
171	K07-100	1	*Verrucomicrobia*	Verrucomicrobia clone Amb_16S_1148 (92.51 %) [EF018510]	Rhizosphere	
172	VE07-01	2	*Actinobacteria*	Actinobacterium clone TR1E4 (99.71 %) [EU117953]	Freshwater	*Candidatus Planktophila limnetica* MWH-EgelM2-3.acI

						(97.82 %) [FJ428831]
173	VE07-02	1	*Unaffiliated*	Clone CK_2C2_2 (82.83 %) [EU488114]	Sediment	
174	VE07-03	1	*Alphaproteobacteria*	Clone TF82 (98.42%) [GU272246]	Rape phyllosphere	*Sphingomonas* sp. PA218 (98.22 %) [AM900781]
175	VE07-05	4	*Betaproteobacteria*	Clone UTFS-OF08-d36-85 (99.57 %) [AB200293]	Sludge	*Hydrogenophaga* sp. EMB 75 (99.78 %) [DQ413154]
176	VE07-07	1	*Bacteroidetes*	*Sphingobacterium* sp. clone ZS-2-83 (99.57 %) [FN668091]	Lake Zurich	*Sediminibacterium* sp. TEGAF015 (93.43 %) [AB470450]
177	VE07-08	1	*Epsilonproteobacteria*	*Arcobacter* sp. clone A1 (99.20 %) [FJ968635]	Microbial fuel cell	*Arcobacter* sp. R-28314 (96.53 %) [AM084114]
178	VE07-10	2	*Actinobacteria*	Actinobacterium clone DE1H8 (99.42 %) [FJ916092]	Temperate lakes	
179	VE07-11	8	*Actinobacteria*	Actinobacterium clone LT1B7 (99.85 %) [EU117819]	Lake epilimnion	
180	VE07-13	1	*Bacteroidetes*	Clone c5LKS101 (96.77 %) [AM086161]	Lake sediment	*Cytophaga* sp. AN-BI4 (88.80 %) [AM157648]
181	VE07-15	2	*Gammaproteobacteria*	Clone Er-MS-95 (96.92 %) [EU542455]	Sediment	*Methylomonas* sp. LW16 (95.93 %) [AF150796]
182	VE07-16	1	*Betaproteobacteria*	Clone 44 (99.27 %) [DQ413103]	Sludge	*Dechloromonas* sp. MissR (96.77 %) [AF170357]
183	VE07-21	1	*Betaproteobacteria*	Clone 231ds5 (94.88 %) [AY212678]	Fecal contamination	*Aquamonas fontana* AQ11 (93.36 %) [AB120965]
184	VE07-23	1	*Actinobacteria*	Actinobacterium clone TW1C9 (99.85 %) [EU117978]	Freshwater	
185	VE07-26	1	*Betaproteobacteria*	Clone 3C003467 (98.93 %) [EU802072]	Chesapeake Bay, MD, USA	*Rhodoferax* sp. Asd M2A1 (98.28 %) [FM955857]
186	VE07-28	1	*Actinobacteria*	Clone T1-57 (95.95 %) [GQ487919]	Soil	
187	VE07-29	1	*Unaffiliated*	Eubacterium WCHB1-58 (90.04 %) [AF050610]	Aquifer	
188	VE07-33	1	*Betaproteobacteria*	Clone DP10.4.16 (95.64 %) [FJ612405]	Dongping Lake Ecosystems	
189	VE07-34	2	*Gammaproteobacteria*	Clone 226ds5 (97.79 %) [AY212673]	Equine Feacal Contamination	*Pseudomonas moorei* Asd MY-A1 (97.63 %) [FM955889]
190	VE07-39	1	*Firmicutes*	Clone R1B-2	Sludge	*Clostridium sticklandii*

				(89.80 %) [FJ167439]		(92.17 %) [M26494]
191	VE07-41	1	*Firmicutes*	Clone HTE5 (99.27 %) [AF418957]	Freshwater reservoir	*Clostridium* sp. P2 (99.25 %) [AY949856]
192	VE07-43	2	*Bacteroidetes*	Clone DP10.4.32 (99.71 %) [FJ612414]	Dongping Lake Ecosystems	
193	VE07-45	1	*Betaproteobacteria*	*Paucibacter toxinivorans* S1030 (97.24 %) [AY515384]	Lake Vihnusjarvi sediment	
194	VE07-48	1	*Gammaproteobacteria*	*Pseudomonas* sp. CJ11064 (99.43 %) [AF500211]		
195	VE07-50	1	*Actinobacteria*	Clone MYS16 (98.72 %) [GU305737]	MiYu Reservoir water	*Solirubrobacter* sp. GSoil 921 (88.84 %) [AB245336]
196	VE07-51	1	*Firmicutes*	Eubacterium WCHB1-71 (96.64 %) [AF050585]	Aquifer	*Pelospora glutarica* WoGl3 (92.60 %) [AJ251214]
197	VE07-52	1	*Betaproteobacteria*	Betaproteobacterium PIB-24 (99.49 %) [AM849435]	Oligomesotrophic lake	*Rhodoferax* sp. Asd M2A1 (97.41 %) [FM955857]
198	VE07-53	1	*Alphaproteobacteria*	Clone 2C229498 (97.70 %) [EU801149]	Delaware Bay, NJ, USA	*Methylocella palustris* Ch3 (96.38 %) [AJ563926]
199	VE07-55	1	*Chloroflexi*	Clone 2C229133 (99.56 %) [EU800895]	Delaware Bay, NJ, USA	
200	VE07-60	4	*Betaproteobacteria*	Clone ZWB5-4 (99.14 %) [FJ801218]	Wetland	*Methylophilus* sp. ECd5 (96.61 %) [AY436794]
201	VE07-61	1	*Chloroflexi*	Chloroflexi clone FNE11-8 (99.34 %) [DQ501314]	Lake water	*Kouleothrix aurantiaca* MYSI-A (86.14 %) [AB079639]
202	VE07-62	1	*Acidobacteria*	Clone 2h-18 (97.46 %) [FJ444648]	Rhizosphere	
203	VE07-63	1	*Actinobacteria*	Clone ML-7-114.2 (96.47 %) [DQ520182]	Shallow hypertrophic freshwater lake	
204	VE07-64	1	*Betaproteobacteria*	Betaproteobacterium clone ZS-4-32 (99.57 %) [FN668031]	Lake Zurich	*Paucibacter* sp. Asd M1A1 (97.70 %) [FM955854]
205	VE07-68	2	*Betaproteobacteria*	Clone HDBW-WB08 (99.49 %) [AB237671]	Aquifer	*Dechloromonas* sp. MissR (97.71 %) [AF170357]
206	VE07-69	11	*Actinobacteria*	Clone MYW3 (99.64 %) [GU305754]	YangHe reservoir/MinYun reservoir	
207	VE07-71	2	*Actinobacteria*	Clone YHW22 (99.49 %) [GU305846]	YangHe reservoir/MinYun reservoir (China)	

208	VE07-72	1	*Actinobacteria*	Actinobacterium clone CB11A12 (98.70 %) [EF471696]	Estuarine bacterioplankton	
209	VE07-73	4	*Actinobacteria*	Actinobacterium clone ZS-4-333 (99.64 %) [FN668241]	Lake Zurich	
210	VE07-75	1	*Alphaproteobacteria*	*Bradyrhizobium* sp. BTAi1 (94.59 %) [CP000494]		
211	VE07-76	1	*Epsilonproteobacteria*	Clone SLB319 (98.41 %) [DQ787688]	Temperate lake sediment	*Sulfuricurvum kujiense* YK-3 (94.81 %) [AB080644]
212	VE07-78	1	*Betaproteobacteria*	Clone sl1354 (98.78 %) [EU244075]	River Leine sediment	*Massilia timonae* CP183-9 (98.57 %) [AJ871463]
213	VE07-79	7	*Betaproteobacteria*	Clone 3C002700 (99.71 %) [EU801416]	Chesapeak Bay, MD, USA	
214	VE07-82	1	*Bacteroidetes*	*Flectobacillus* sp. clone ZS-2-89 (99.78 %) [FN668109]	Lake Zurich	*Arcicella* sp. MG83 (93.38 %) [AJ746140]
215	VE07-85	2	*Unaffiliated*	Clone YHY15 (99.85 %) [GU305800]	Shallow hypertrophic lake	
216	VE07-89	1	*Alphaproteobacteria*	Clone DP10.4.4 (99.63 %) [FJ612398]	Dongping Lake Ecosystems	
217	VE07-90	1	*Alphaproteobacteria*	Clone DP7.3.104 (98.74 %) [FJ612228]	Dongping Lake Ecosystems	*Roseomonas* sp. SK 65 (96.13 %) [AM991117]
218	VE07-92	1	*Firmicutes*	Clone HH_aai36f02 (95.18 %) [EU459333]	Mammal gut	*Epulopiscium* sp. N.l._1__5 (93.35 %) [AY844964]
219	VE07-96	5	*Actinobacteria*	Clone MYS21 (99.78 %) [GU305741]	YangHe reservoir/MinYun reservoir	
**2008**						
220	A08-102	2	*Chloroflexi*	Crater Lake clone CL500-48 (99.12 %) [AF316757]	Oligotrophic Crater Lake water	
221	A08-106	1	*Unaffiliated*	Clone 2C228453 (85.33 %) [EU800378]	Delaware Bay, NJ, USA	
222	A08-107	1	*Betaproteobacteria*	Proteobacterium clone F13_2C_FL (97.92 %) [EF682968]	African dust	*Herbaspirillum* sp. GT-L15 (97.75 %) [GQ355286]
223	A08-108	6	*Actinobacteria*	Clone MYY14 (99.27 %) [GU305708]	YangHe reservoir/MinYun reservoir	
224	A08-109	2	*Planctomycetes*	Clone DP7.3.10 (95.16 %) [FJ612210]	Dongping Lake Ecosystems	Planctomycete GMD21C08 (84.63%) [AY162119]

225	A08-113	2	*Gammaproteobacteria*	*Pseudoxanthomonas mexicana* AMX 26B (91.52 %) [AF273082]	UASB reactor sludge	
226	A08-119	1	*Alphaproteobacteria*	Clone D44 35 (99.70 %) [EU580491]	Lake Constance	*Sphingomonas* sp. B18 (99.85 %) [AF410927]
227	A08-121	1	*Bacteroidetes*	Bacteroidetes cloneAS30 (98.18 %) [EU283361]	Wastewater	*Tuber borchii* symbiont b-17BO (95.11 %) [AF070444]
228	A08-122	3	*Planctomycetes*	Clone DP10.2.53 (94.38 %) [FJ612357]	Dongping Lake Ecosystems	
229	A08-123	3	*Bacteroidetes*	Clone 3C003304 (99.71 %) [EU801923]	Chesapeak Bay, MD, USA	*Owenweeksia hongkongensis* (88.80 %) [AB125062]
230	A08-125	1	*Bacteroidetes*	Clone SING480 (99.42 %) [HM129126]	Tibetan Lake (China)	*Sediminibacterium* sp. TEGAF015 (93,86 %) [AB470450]
231	A08-126	1	*Gammaproteobacteria*	Clone S25_35 (95.65 %) [EF573691]	Coco's island	*Coxiella burnetii* CbuK_Q154 (87.20 %) [CP001020]
232	A08-127	8	*Actinobacteria*	Clone MYW3 (99.78 %) [GU305754]	YangHe reservoir/MinYun reservoir	
233	A08-131	1	*Unaffiliated*	Clone ML-9-34.2 (96.55 %) [DQ520188]	Hypertrophic lake	
234	A08-132	1	*Bacteroidetes*	Clone MYY7 (95.86 %) [GU305702]	YangHe reservoir/MinYun reservoir	
235	A08-134	1	*Chloroflexi*	Clone MYS10 (99.48 %) [GU305734]	YangHe reservoir/MinYun reservoir	
236	A08-135	1	*Betaproteobacteria*	Clone FA02B02 (99.34 %) [FM872672]	House dust	*Massilia brevitalea* byr23-80 (98.77 %) [EF546777]
237	A08-136	2	*Bacteroidetes*	Clone 2C229371 (99.78 %) [EU801066]	Delaware Bay, NJ, USA	*Arcicella* sp. MG83 (93.66 %) [AJ746140]
238	A08-137	1	*Betaproteobacteria*	Clone ML-5-90.2 (99.86 %) [DQ520167]	Shallow hypertrophic lake	*Variovorax* sp. EMB 263 (97.89 %) [DQ413166]
239	A08-139	1	*Planctomycetes*	Clone K0029 (97.04 %) [FJ820433]	Wuliangsuhai Lake (China)	
240	A08-140	1	*Alphaproteobacteria*	*Sphingomonas dokdonensis* DS-4 (99.17 %) [DQ178975]	Soil	
241	A08-141	3	*Actinobacteria*	Clone MYS21 (99.85 %) [GU305741]	YangHe reservoir/MinYun reservoir	

242	A08-142	10	*Unaffiliated*	SAR11 cluster Alphaproteobacterium clone ZS-4-50 (99.48 %) [FN665765]	Temperate lakes	
243	A08-148	1	*Unaffiliated*	Clone 6S1-13 (99.12 %) [GU208248]	Dongping Lake sediment	
244	A08-150	1	*Alphaproteobacteria*	Clone A-35 (99.63 %) [GQ472125]	Algicidal enrichment culture	*Rhizobium* sp. W3 (99.33 %) [EU781656]
245	A08-153	1	*Alphaproteobacteria*	Alphaproteobacterium clone Xbod17 (92.99 %) [GU129938]	Marine worm	*Phaeospirillum fulvum* S3 (91.54 %) [AF508113]
246	A08-155	1	*Actinobacteria*	Actinobacterium clone LA1C3 (98.84 %) [FJ916093]	Temperate lakes	*Actinomycetales* bacterium GSoil 1632 (89.76 %) [AB245397]
247	A08-156	1	*Actinobacteria*	Clone 5C231028 (99.42 %) [EU803457]	Lake Gatun, Panama	
248	A08-159	1	*Actinobacteria*	Clone 3C003327 (99.78 %) [EU801945]	Chesapeak Bay, MD, USA	
249	A08-160	3	*Bacteroidetes*	*Sphingobacterium* sp. clone ZS-2-342 (98.51 %) [FN668080]	Lake Zurich	*Terrimonas lutea* (95.54 %) [AB192292]
250	A08-163	4	*Unaffiliated*	Clone IC-33 (97.09 %) [AB255061]	Corroding concrete	
251	A08-165	1	*Alphaproteobacteria*	*Afipia broomeae* F186 (99.70 %) [U87759]		
252	A08-166	1	*Gammaproteobacteria*	*Legionella*-like amoebal pathogen HT99 (93.11 %) [AY741401]	Hot tub	*Ectothiorhodospira* sp. AM4 (90.16 %) [EU252492]
253	A08-171	5	*Bacteroidetes*	Clone DP10.4.3 (98.49 %) [FJ612397]	Dongping Lake Ecosystems	
254	B08-101	1	*Cyanobacteria*	Clone DP3.2.22 (99.18 %) [FJ612143]	Dongping Lake Ecosystems	*Thalassiosira pseudonana* - chloroplast (98.59 %) [EF067921]
255	B08-102	4	*Actinobacteria*	Actinobacterium clone CH1D5 (99.85 %) [EU117613]	Lake epilimnion	
256	B08-103	1	*Actinobacteria*	Actinobacterium clone 79 T12d-oil (94.93 %) [FM242392]	Sediment	*Candidatus Microthrix calida* TNO2-1 (90.59 %) [DQ147277]
257	B08-104	2	*Planctomycetes*	Clone DP10.2.53 (94.24 %) [FJ612357]	Dongping Lake Ecosystems	
258	B08-105	1	*Bacteroidetes*	Flexibacteraceae bacterium 1351 (97.08%)	Soil	*Hymenobacter* sp.

				[EU543663]		SAFR-033 (92.99%) [AY167829]
259	B08-107	8	*Actinobacteria*	Clone MYW3 (99.64 %) [GU305754]	YangHe reservoir/MinYun reservoir	
260	B08-108	2	*Bacteroidetes*	Clone 3C003304 (99.93 %) [EU801923]	Chesapeake Bay, MD, USA	*Owenweeksia hongkongensis* (88.94 %) [AB125062]
261	B08-114	1	*Gammaproteobacteria*	Clone DP10.3.24 (95.96 %) [FJ612378]	Dongping Lake Ecosystems	
262	B08-115	1	*Bacteroidetes*	Bacteroidetes clone XZXXH108 (96.85 %) [EU703423]	Tibetan lakes	*Coccinistipes vermicola* IMCC1411 (84.95 %) [EF108212]
263	B08-116	1	*Actinobacteria*	Actinobacterium clone CB11A12 (99.20 %) [EF471696]	Estuarine bacterioplankton	
264	B08-118	1	*Deltaproteobacteria*	Clone S11-22 (89.12 %) [EU287205]	Surface sediment (Pacific Arctic Ocean)	*Desulforhopalus vacuolatus* (83.77 %) [L42613]
265 C	B08-120	1	*Bacteroidetes*	*Sphingobacterium* sp. clone ZS-2-342 (98.65 %) [FN668080]	Lake Zurich	*Terrimonas lutea* (94.06 %) [AB192292]
266	B08-123	2	*Bacteroidetes*	Sphingobacteriales clone SF07 (98,56 %) [AJ697707]	Mesotrophic lake	*Candidatus Aquirestis calciphila* MS-Falk1-L (98.39 %) [AJ786341]
267	B08-127	9	*Alphaproteobacteria*	Clone DP10.4.4 (99.41 %) [FJ612398]	Dongping Lake Ecosystems	
268	B08-137	1	*Deltaproteobacteria*	Clone 5C231108 (96.12 %) [EU803523]	Aquatic Bacterial Communities	
269	B08-138	2	*Actinobacteria*	Clone MYS21 (99.56 %) [GU305741]	YangHe reservoir/MinYun reservoir	
270	B08-139	3	*Actinobacteria*	Clone 5C230997 (95.39 %) [EU803430]	Lake Gatun, Panama	
271	B08-146	6	*Actinobacteria*	Actinobacterium clone ZS-2-309 (99.13 %) [FN668222]	Lake Zurich	*Candidatus Planktophila limnetica* MWH-EgelM2-3.acI (97.31 %) [FJ428831]
272	B08-148	3	*Bacteroidetes*	Clone K2-30-6 (95.14 %) [AY344418]	Hawaiian archipelago	
273	B08-149	3	*Alphaproteobacteria*	Clone S25_718 (89.70 %) [EF574374]	Coco's island	*Filomicrobium insigne* SLG5B-19

						(85.00 %) [EF117253]
274	B08-151	1	*Actinobacteria*	Clone D19 (99.49 %) [EU234320]	Wang Yang River	
275	B08-154	2	*Actinobacteria*	Actinobacteria clone QEDV2AC09 (97.17 %) [CU919363]	Sludge	*Cellulomonas* sp. F11 (90.98 %) [EU697083]
276	B08-158	1	*Bacteroidetes*	Clone DP10.4.3 (98.42 %) [FJ612397]	Dongping Lake Ecosystems	
277	K08-101	1	*Alphaproteobacteria*	*Bradyrhizobium* sp. SOD34 (99.48 %) [EF125933]		
278	K08-102	2	*Bacteroidetes*	Clone 3C003304 (99.45 %) [EU801923]	Chesapeak Bay, MD, USA	*Owenweeksia hongkongensis* (88.60 %) [AB125062]
279	K08-104	16	*Alphaproteobacteria*	Clone DP10.4.4 (99.93 %) [EU803730]	Lake Gatun, Panama	
280	K08-106	4	*Actinobacteria*	Clone MYW3 (99.64 %) [GU305754]	YangHe reservoir/MinYun reservoir	
281	K08-107	3	*Actinobacteria*	Clone MYS21 (99.71%) [GU305741]	YangHe reservoir/MinYun reservoir	
282	K08-108	2	*Planctomycetes*	Clone DP10.2.53 (94.59 %) [FJ612357]	Dongping Lake Ecosystems	
283	K08-109	3	*Betaproteobacteria*	*Curvibacter* putative symbiont of *Hydra magnipapillata* genomic scaffold HmaUn_WGA71069_1 (99.35 %) [FN543107]	Symbiont of *Hydra magnipapillata*	*Aquamonas fontana* AQ11 (98.42 %) [AB120965]
284	K08-110	1	*Alphaproteobacteria*	Clone DP7.3.104 (99.18 %) [FJ612228]	Dongping Lake Ecosystems	*Roseomonas* sp. SK 65 (96.35 %) [AM991117]
285	K08-112	1	*Planctomycetes*	Clone nbw428a05c1 (94.66 %) [GQ094017]	Human skin microbiome	Planctomycete isolate A-2 (81.03 %) [AM056027]
286	K08-113	1	*Bacteroidetes*	Clone DP7.5.43 (97.93 %) [FJ612299]	Dongping Lake Ecosystems	
287	K08-114	5	*Bacteroidetes*	Clone 2C229371 (99.64 %) [EU801066]	Delaware Bay, NJ, USA	*Arcicella* sp. MG83 (93.06 %) [AJ746140]
288	K08-115	2	*Verrucomicrobia*	Clone MYW29 (98.79 %) [GU305773]	YangHe reservoir/MinYun reservoir	*Methylacidiphilum infernorum* V4 CP000975 (84 %) [CP000975]
289	K08-116	1	*Verrucomicrobia*	Clone aab56b01 (95.05 %) [DQ814962]	Adult zebrafish digestive tract	*Luteolibacter pohnpeiensis* A4T-83 (89.51 %) [AB331895]
290	K08-117	3	*Chloroflexi*	Crater Lake clone CL500-48	Oligotrophic Crater lake	

				(99.41 %) [AF316757]	water	
291	K08-118	1	*Planctomycetes*	Crater Lake clone CL500-3 (93.71 %) [AF316767]	Oligotrophic Crater lake water	
292	K08-120	1	*Gammaproteobacteria*	Clone MC1_16S_55 (98.99 %) [EU662624]	Sulfidic water, Movile Cave, Romania	*Rheinheimera chironomi* K19414 (98.12%) [DQ298025]
293	K08-122	4	*Actinobacteria*	Actinobacterium clone ZS-4-333 (97.90 %) [FN668241]	Lake Zurich	
294	K08-123	1	*Betaproteobacteria*	Clone DP10.4.16 (97.60 %) [FJ612405]	Dongping Lake Ecosystems	*Polynucleobacter acidiphobus* MWH-IPGL7W22 (97.55%) [AJ876403]
295	K08-125	1	*Bacteroidetes*	Sphingobacteriales clone SF68 (98.35 %) [AJ697704]	Mesotrophic lake	*Algoriphagus* sp. RC-layer4 (95.78 %) [EU677424]
296	K08-127	1	*Actinobacteria*	Clone SINI1012 (96.27 %) [HM126680]	Chaerhan Lake (China)	*Kaistibacter ginsenosidimutans* BXN7-7 (89.50 %) [EU423306]
297	K08-128	1	*Gammaproteobacteria*	Gammaproteobacterium clone GPTSA100-21 (97.06 %) [DQ914844]	Spring (India)	*Rheinheimera* sp. G2DM-88 (95.78 %) [EU037269]
298	K08-142	1	*Alphaproteobacteria*	Clone DP7.5.111 (92.96 %) [FJ612309]	Dongping Lake Ecosystems	*Azorhizobium caulinodans* ORS 571 (83.99 %) [AP009384]
299	K08-144	1	*Bacteroidetes*	Sphingobacterium sp. clone ZS-2-342 (99.29 %) [FN668080]	Lake Zurich	*Terrimonas lutea* (94.70 %) [AB192292]
300	K08-148	1	*Actinobacteria*	Clone 3C002490 (87.09 %) [EU801240]	Chesapeak Bay, MD, USA	*Friedmanniella spumicola* (84.95 %) [AF062535]
301	K08-150	1	*Actinobacteria*	Actinobacterium clone CB11A12 (99.20 %) [EF471696]	Estuarine bacterioplankton	
302	VE08-102	1	*Actinobacteria*	*Blastococcus* sp. BC521 (99.12 %) [AJ316573]		
303	VE08-104	2	*Betaproteobacteria*	Clone 015B-E09 (98.57 %) [AY662039]	Groundwater	*Siderooxidans lithoautotrophicus* ES-1 (95.51 %) [DQ386264]

304	VE08-105	10	*Actinobacteria*	Actinobacterium clone ZS-2-02 (99.78 %) [FN668289]	Lake Zurich	
305	VE08-108	1	*Bacteroidetes*	Clone DP7.4.54 (95.35 %) [FJ612278]	Dingpong Lake Ecosystems	*Hymenobacter* sp. 29F (87.77%) [AY647897]
306	VE08-109	4	*Chloroflexi*	Clone 2C229133 (99.48 %) [EU800895]		
307	VE08-110	6	*Unaffiliated*	Eubacterium WCHB1-58 (88.24 %) [AF050610]	Aquifer	
308	VE08-113	1	*Cyanobacteria*	Clone DP7.1.5 (97.99 %) [FJ612231]	Dongping Lake Ecosystems	*Coscinodiscus radiatus* - chlroplast p148 (88.41 %) [AJ536462]
309	VE08-114	1	*Alphaproteobacteria*	Alphaproteobacterium clone TK-NH3 (99.18 %) [DQ463742]	Tanganyika Lake	*Sphingomonas* sp. CC-LTT-6 (99.18 %) [AY941088]
310	VE08-115	1	*Planctomycetes*	Clone MYS7 (99.27 %) [GU305732]	YangHe reservoir/MinYun reservoir	*Planctomycete* str. 535 (92.64 %) [AJ231176]
311	VE08-118	1	*Betaproteobacteria*	Clone 2_G8 (98.50 %) [EU589321]	Soil	*Methylophilus* sp. enrichment culture MWE_C44 (98.28 %) [FJ391507]
312	VE08-120	1	*Bacteroidetes*	*Sphingobacterium* sp. clone ZS-2-83 (99.64 %) [FN668091]	Lake Zurich	*Sediminibacterium* sp. TEGAF015 (93.51 %) [AB470450]
313	VE08-121	2	*Betaproteobacteria*	Clone S25_868 (99.35 %) [EF574524]	Coco's island	*Limnobacter thiooxidans* CS-K2 (98.99 %) [AJ289885]
314	VE08-123	3	*Planctomycetes*	Planctomycete clone CY0ARA030H03 (97.37 %) [BX294821]	Anaerobic digester	*Planctomyces* sp. (599) (87.12 %) [AJ231189]
315	VE08-126	4	*Betaproteobacteria*	Betaproteobacterium clone ADK-CSe02-53 (99.42 %) [EF520468]	Acid impacted lake	
316	VE08-129	1	*Planctomycetes*	*Singulisphaera* sp. clone 2h-7 (97.76%) [FJ444639]	Rhizosphere	*Singulisphaera acidiphila* MPL1015 (96.97 %) [AM902525]
317	VE08-130	1	*Firmicutes*	Clone SS_WC_22 (89.96 %) [FJ973592]	Lake	*Acetivibrio cellulolyticus* (ATCC 33288) (87.28 %) [L35516]

318	VE08-131	3	*Planctomycetes*	Clone ambient_uncontrolled-78 (96.36 %) [GU454939]	Sludge	*P. limnophilus* (88.22 %) [X62911]
319	VE08-133	1	*Actinobacteria*	Actinobacterium clone ZS-2-309 (99.20 %) [FN668222]	Lake Zurich	*Candidatus Planktophila limnetica* MWH-EgelM2-3.acI (97.38%) [FJ428831]
320	VE08-134	4	*Actinobacteria*	Clone MYS21 (99.78%) [GU305741]	YangHe reservoir/MinYun reservoir	
321	VE08-135	1	*Bacteroidetes*	Clone MYY7 (96.08 %) [GU305702]	YangHe reservoir/MinYun reservoir	
322	VE08-138	1	*Betaproteobacteria*	Clone 3BH-10FF (99.35 %) [EU937983]	Freshwater environment	*Rhodoferax* sp. Asd M2A1 (99.14 %) [FM955857]
323	VE08-140	1	*Alphaproteobacteria*	Clone FGL7S_B51 (97.36 %) [FJ437985]	Fayetteville Green Lake (NY)	*Sphingobacterium* sp. P-17 (91.46 %) [AM411963]
324	VE08-141	1	*Alphaproteobacteria*	Alphaproteobacterium clone F0813 (99.41 %) [AF235997]		*Sphingomonas* sp. B18 (96.95 %) [AF410927]
325	VE08-144	2	*Bacteroidetes*	Sphingobacteriales clone SF68 (99.21 %) [AJ697704]	Mesotrophic lake	*Algoriphagus aquatilis* A8-7 (97.28 %) [EU313811]
326	VE08-145	1	*Actinobacteria*	Actinobacterium clone DE1H8 (98.91 %) [FJ916092]	Temperate lake	
327	VE08-147	1	*Bacteroidetes*	Clone K2-30-6 (95.14 %) [AY344418]	Hawaiian archipelago	
328	VE08-148	1	*Betaproteobacteria*	Clone DP10.4.6 (99.50%) [FJ612399]	Dongping Lake Ecosystems	*Pandoraea* sp. Y1 (93%) [AB222022]
329	VE08-149	4	*Actinobacteria*	Actinobacterium clone LA1C3 (99.20 %) [FJ916093]	temperate lakes	*Actinomycetales* bacterium GSoil 1632 (90.19 %) [AB245397]
330	VE08-150	1	*Actinobacteria*	Clone D19 (99.71 %) [EU234320]	Wang Yang River	
331	VE08-152	2	*Chloroflexi*	Clone SRRB40 (94.98 %) [AB240513]	Rhizosphere	
332	VE08-154	1	*Actinobacteria*	Actinobacterium clone LT1B7 (99.98 %) [EU117819]	Freshwater	
333	VE08-155	2	*Alphaproteobacteria*	Clone S25_718 (89.84 %) [EF574374]	Coco's island	*Filomicrobium insigne* SLG5B-19

					(85.15 %) [EF117253]
334	VE08-160	1	*Betaproteobacteria*	Clone DP10.4.32 (99.79 %) [FJ612414]	Dongping Lake Ecosystems
335	VE08-161	1	*Betaproteobacteria*	Clone B67 (98.21 %) [FJ660535]	Activated sludge
336	VE08-162	1	*Actinobacteria*	Actinobacterium clone TW1B6 (99.78 %) [EU117973]	Lake epilimnion
337	VE08-163	1	*Alphaproteobacteria*	*Brevundimonas vesicularis* Asd M7-3 (99.55 %) [FM955876]	Glacier

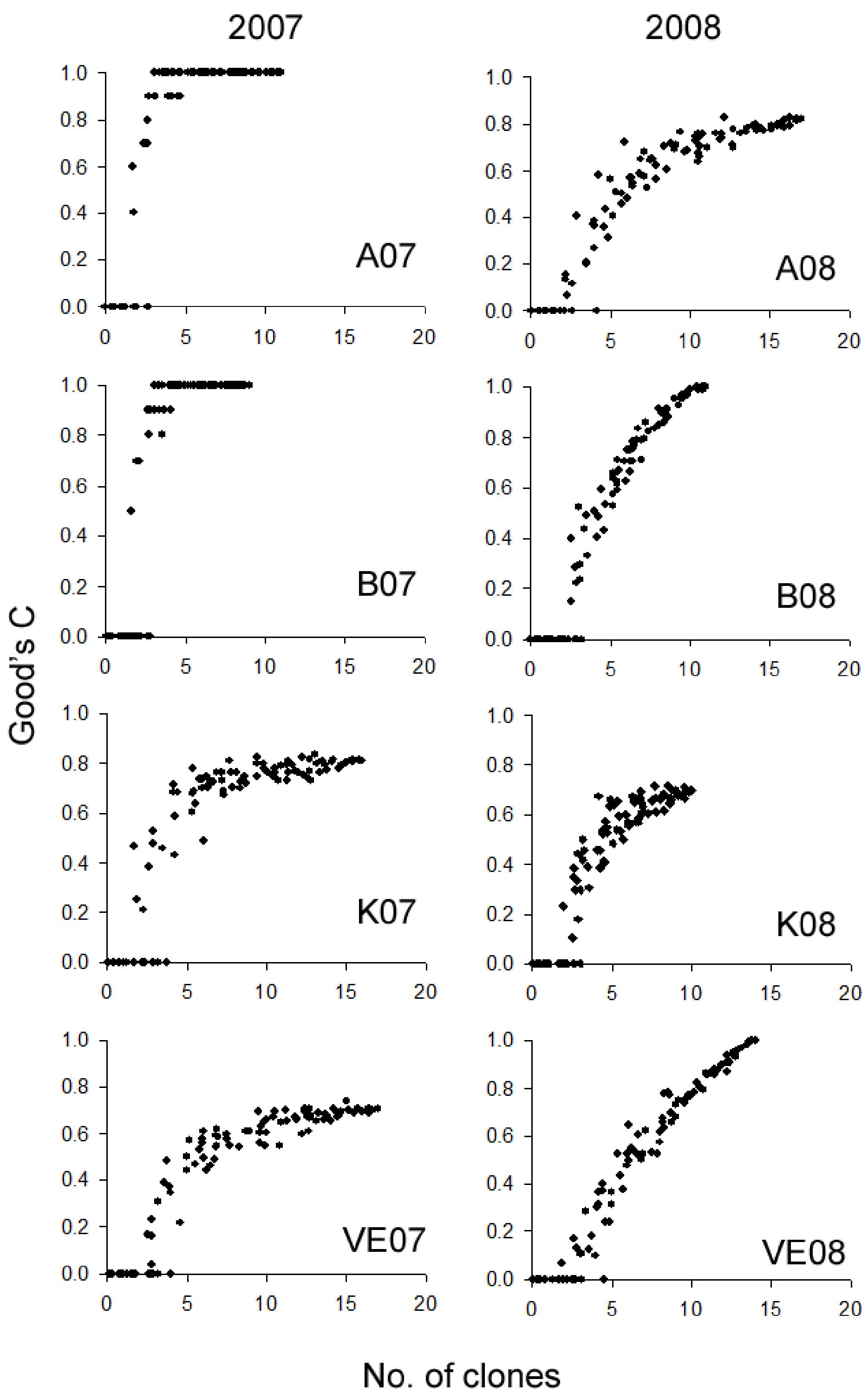
2007
2008
A07
A08
B07
B08
K07
K08
VE07
VE08
Good's C
No. of clones
0.0
0.2
0.4
0.6
0.8
1.0
0
5
10
15
20

Fig. S1A.

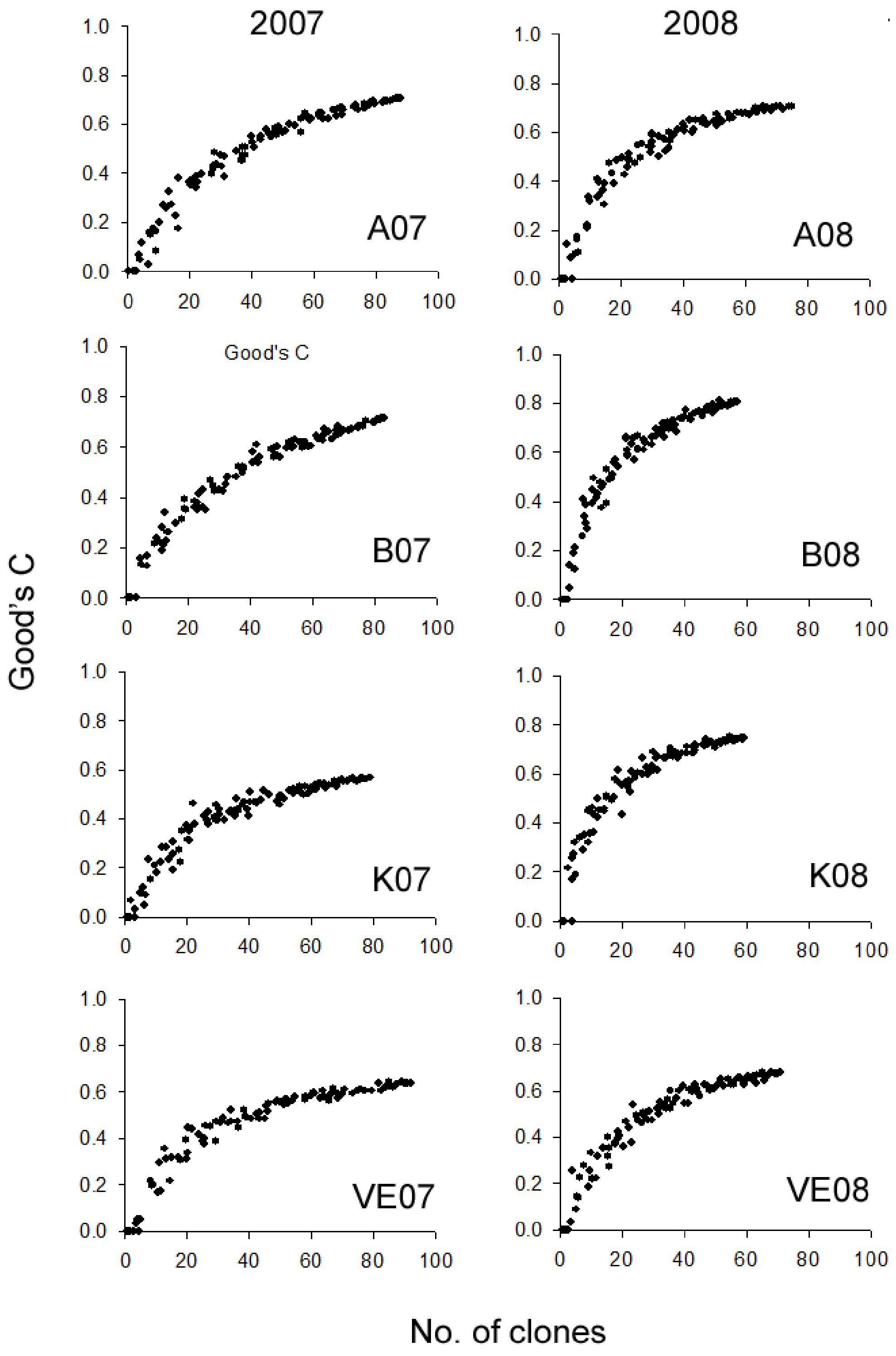

Fig. S1B.

Fig. S1. Archaeal (A) and bacterial (B) clone library coverage based on Good's C estimator from the water column of Lake Marathonas, Greece.

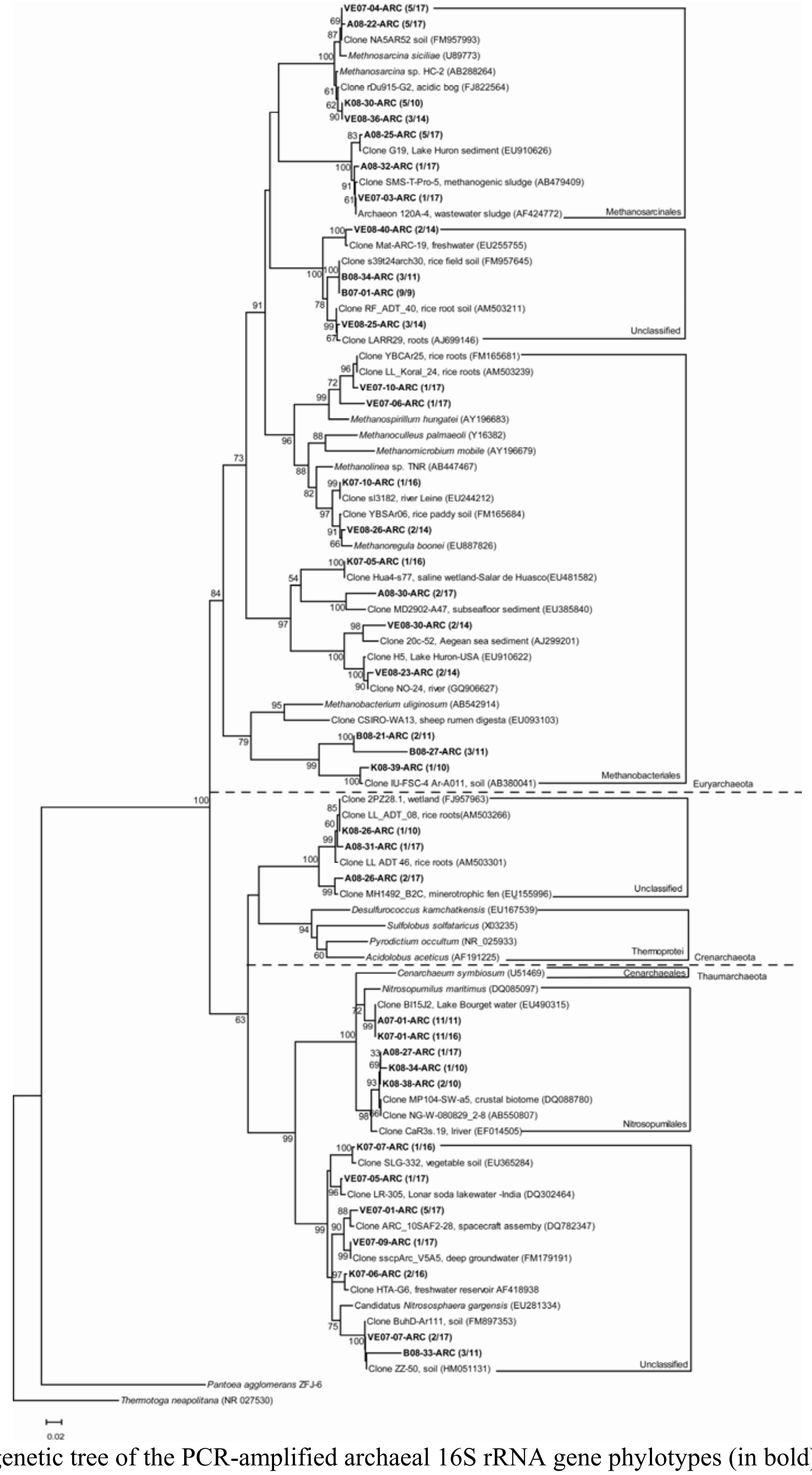

Fig. S2. Phylogenetic tree of the PCR-amplified archaeal 16S rRNA gene phylotypes (in bold) (ca. 570 bp) in the water column of Marathonas Reservoir, Greece, based on the neighbour-joining method as determined by distance using Kimura's two-parameter correction. Numbers of identical (≥98% sequence similarity) phylotypes of the total number phylotypes found in each station are shown in parentheses. One thousand bootstrap analyses (distance) were conducted, and percentages ≥50% are indicated at nodes. Numbers in brackets are GenBank accession numbers. Scale bar represents 2% estimated distance.

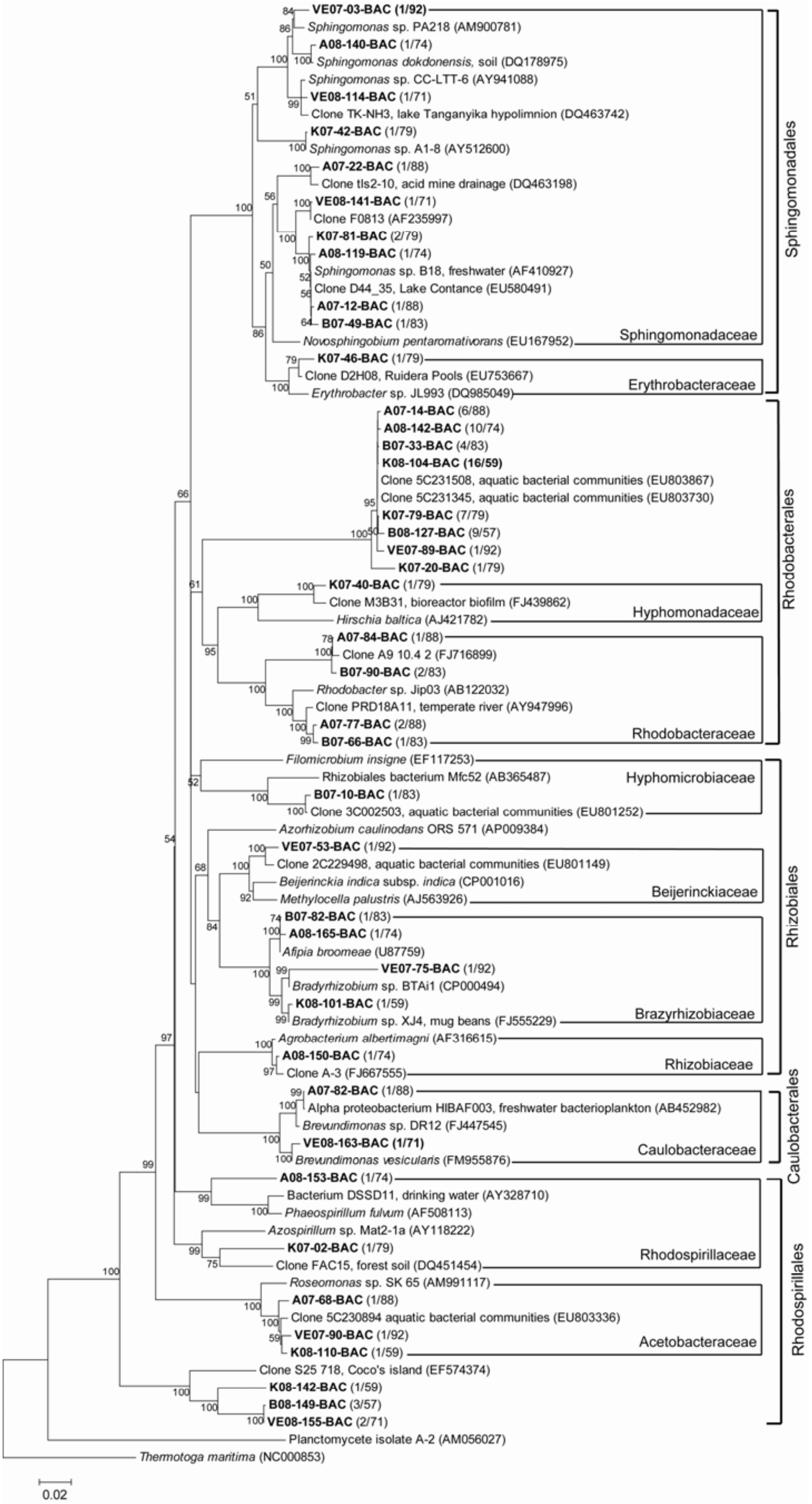

Fig. S3. Phylogenetic tree of the PCR-amplified 16S rRNA gene phylotypes (in bold) (ca. 1390 bp) of the Alphaproteobacteria in the water column of Marathonas Reservoir, Greece, based on the neighbour-joining method as determined by distance using Kimura's two-parameter correction. Numbers of identical (≥98% sequence similarity) phylotypes of the total number phylotypes found in each station are shown in parentheses. One thousand bootstrap analyses (distance) were conducted, and percentages ≥50% are indicated at nodes. Numbers in brackets are GenBank accession numbers. Scale bar represents 2% estimated distance.

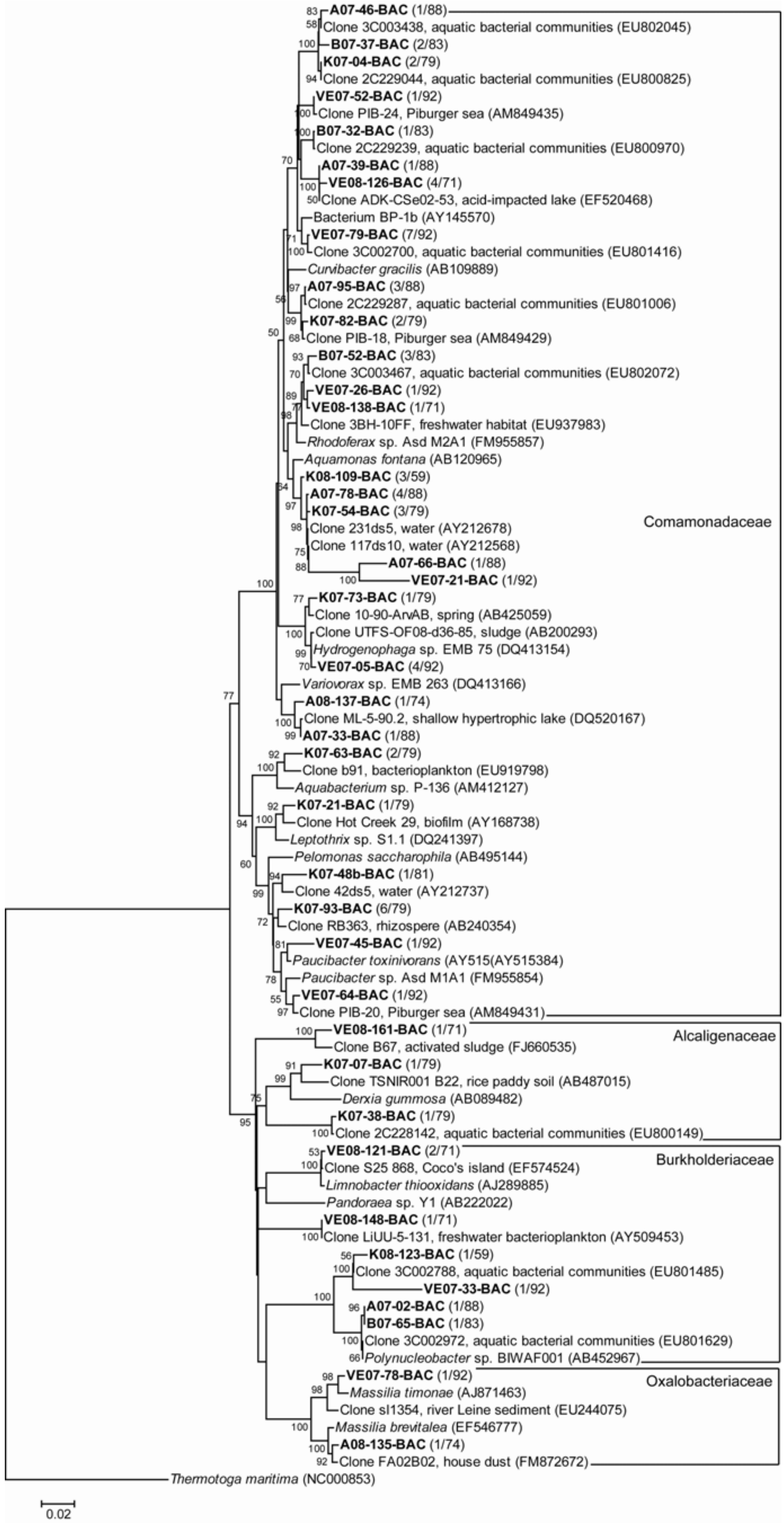

Fig. S4. Phylogenetic tree of the PCR-amplified 16S rRNA gene phylotypes (in bold) (ca. 1390 bp) of the rest of the order Burkholderiales (Betaproteobacteria) in the water column of Marathonas Reservoir, Greece, based on the neighbour-joining method as determined by distance using Kimura's two-parameter correction. Numbers of identical (≥98% sequence similarity) phylotypes of the total number phylotypes found in each station are shown in parentheses. One thousand bootstrap analyses (distance) were conducted, and percentages ≥50% are indicated at nodes. Numbers in brackets are GenBank accession numbers. Scale bar represents 2% estimated distance.

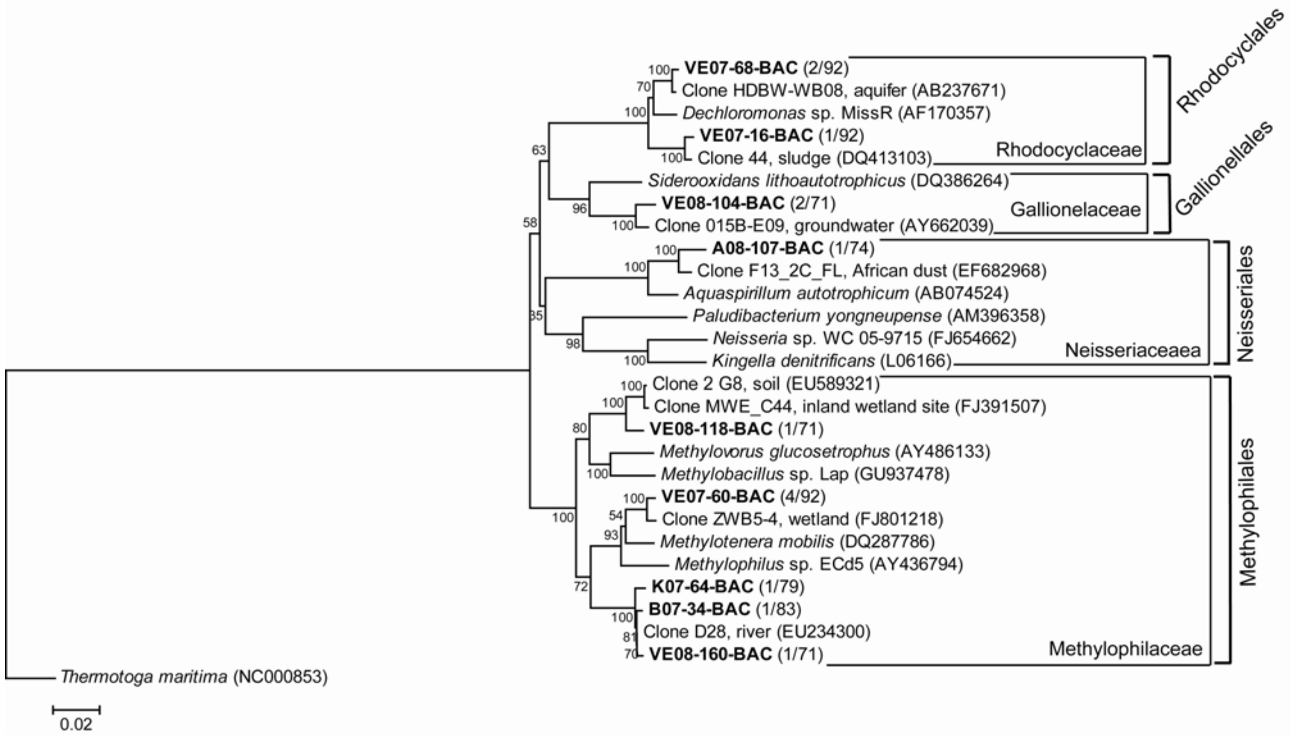

Fig. S5. Phylogenetic tree of the PCR-amplified 16S rRNA gene phylotypes (in bold) (ca. 1390 bp) of the rest of the Betaproteobacteria in the water column of Marathonas Reservoir, Greece, based on the neighbour-joining method as determined by distance using Kimura's two-parameter correction. Numbers of identical (≥98% sequence similarity) phylotypes of the total number phylotypes found in each station are shown in parentheses. One thousand bootstrap analyses (distance) were conducted, and percentages ≥50% are indicated at nodes. Numbers in brackets are GenBank accession numbers. Scale bar represents 2% estimated distance.

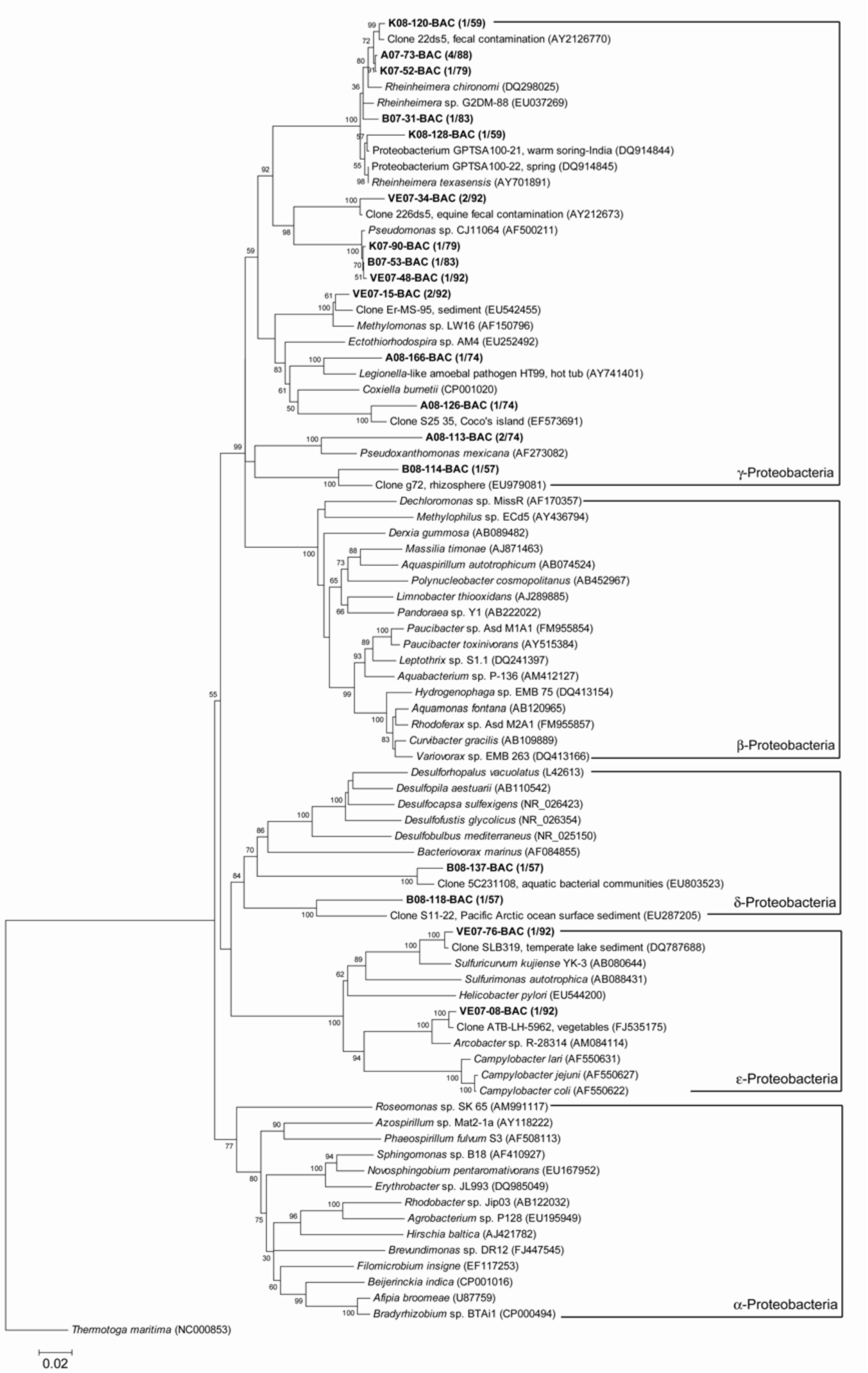

Fig. S6. Phylogenetic tree of the PCR-amplified 16S rRNA gene phylotypes (in bold) (ca. 1390 bp) of α-, γ-, δ- and Epsilonproteobacteria in the water column of Marathonas Reservoir, Greece, based on the neighbour-joining method as determined by distance using Kimura's two-parameter correction. Numbers of identical (≥98% sequence similarity) phylotypes of the total number phylotypes found in each station are shown in parentheses. One thousand bootstrap analyses (distance) were conducted, and percentages ≥50% are indicated at nodes. Numbers in brackets are GenBank accession numbers. Scale bar represents 2% estimated distance.

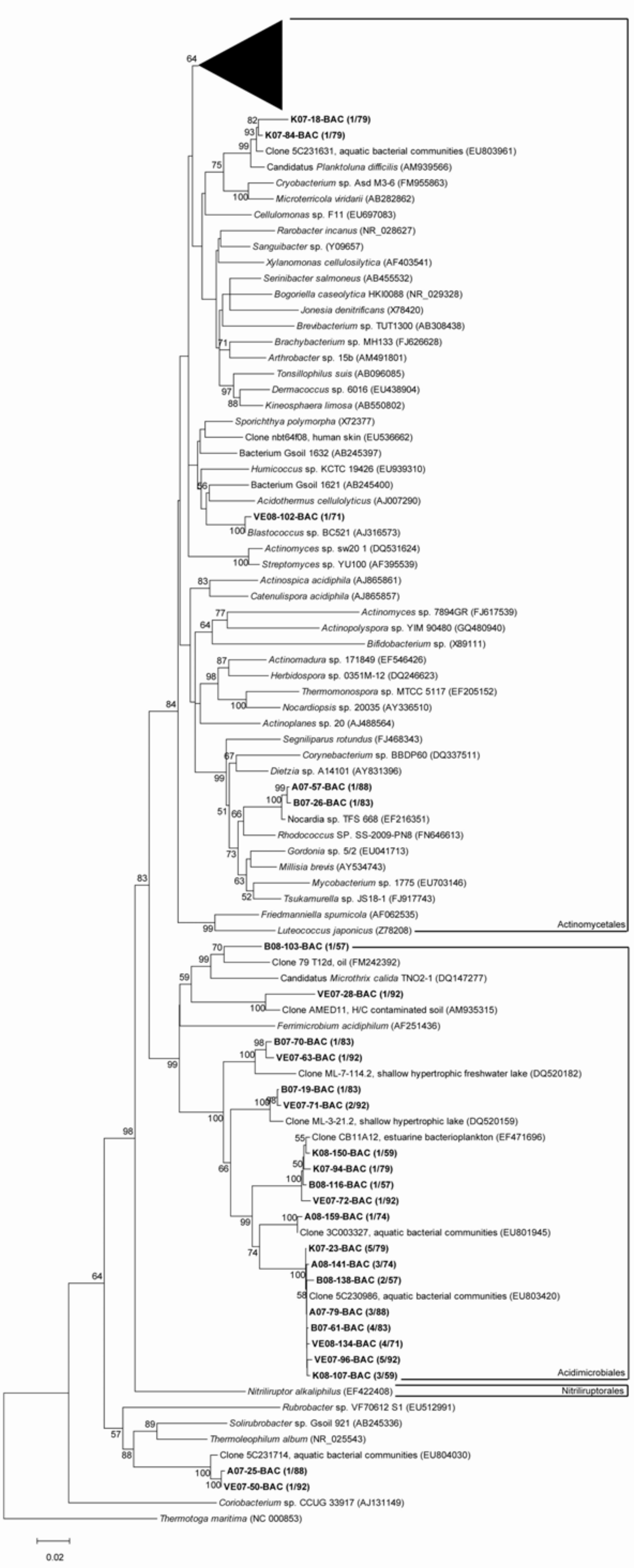
64
82
K07-18-BAC (1/79)
93
K07-84-BAC (1/79)
99
Clone 5C231631, aquatic bacterial communities (EU803961)
75
Candidatus Planktoluna difficilis (AM939566)
Cryobacterium sp. Asd M3-6 (FM955863)
100
Microterricola viridarii (AB282862)
Cellulomonas sp. F11 (EU697083)
Rarobacter incanus (NR_028627)
Sanguibacter sp. (Y09657)
Xylanomonas cellulosilytica (AF403541)
Serinibacter salmoneus (AB455532)
Bogoriella caseolytica HKI0088 (NR_029328)
Jonesia denitrificans (X78420)
Brevibacterium sp. TUT1300 (AB308438)
71
Brachybacterium sp. MH133 (FJ626628)
Arthrobacter sp. 15b (AM491801)
Tonsillophilus suis (AB096085)
97
Dermacoccus sp. 6016 (EU438904)
88
Kineosphaera limosa (AB550802)
Sporichthya polymorpha (X72377)
Clone nbt64f08, human skin (EU536662)
Bacterium Gsoil 1632 (AB245397)
Humicoccus sp. KCTC 19426 (EU939310)
56
Bacterium Gsoil 1621 (AB245400)
Acidothermus cellulolyticus (AJ007290)
VE08-102-BAC (1/71)
100
Blastococcus sp. BC521 (AJ316573)
Actinomyces sp. sw20 1 (DQ531624)
100
Streptomyces sp. YU100 (AF395539)
83
Actinospica acidiphila (AJ865861)
Catenulispora acidiphila (AJ865857)
77
Actinomyces sp. 7894GR (FJ617539)
64
Actinopolyspora sp. YIM 90480 (GQ480940)
Bifidobacterium sp. (X89111)
87
Actinomadura sp. 171849 (EF546426)
98
Herbidospora sp. 0351M-12 (DQ246623)
Thermomonospora sp. MTCC 5117 (EF205152)
100
Nocardiopsis sp. 20035 (AY336510)
84
Actinoplanes sp. 20 (AJ488564)
Segniliparus rotundus (FJ468343)
67
Corynebacterium sp. BBDP60 (DQ337511)
Dietzia sp. A14101 (AY831396)
99
99
A07-57-BAC (1/88)
100
B07-26-BAC (1/83)
51
66
Nocardia sp. TFS 668 (EF216351)
Rhodococcus SP. SS-2009-PN8 (FN646613)
73
Gordonia sp. 5/2 (EU041713)
Millisia brevis (AY534743)
63
Mycobacterium sp. 1775 (EU703146)
52
Tsukamurella sp. JS18-1 (FJ917743)
83
Friedmanniella spumicola (AF062535)
99
Luteococcus japonicus (Z78208)
Actinomycetales
70
B08-103-BAC (1/57)
99
Clone 79 T12d, oil (FM242392)
59
Candidatus Microthrix calida TNO2-1 (DQ147277)
VE07-28-BAC (1/92)
100
Clone AMED11, H/C contaminated soil (AM935315)
Ferrimicrobium acidiphilum (AF251436)
98
B07-70-BAC (1/83)
100
VE07-63-BAC (1/92)
99
Clone ML-7-114.2, shallow hypertrophic freshwater lake (DQ520182)
B07-19-BAC (1/83)
100
98
VE07-71-BAC (2/92)
100
Clone ML-3-21.2, shallow hypertrophic lake (DQ520159)
98
55
Clone CB11A12, estuarine bacterioplankton (EF471696)
K08-150-BAC (1/59)
50
K07-94-BAC (1/79)
66
100
B08-116-BAC (1/57)
VE07-72-BAC (1/92)
100
A08-159-BAC (1/74)
99
Clone 3C003327, aquatic bacterial communities (EU801945)
74
K07-23-BAC (5/79)
A08-141-BAC (3/74)
100
B08-138-BAC (2/57)
64
58
Clone 5C230986, aquatic bacterial communities (EU803420)
A07-79-BAC (3/88)
B07-61-BAC (4/83)
VE08-134-BAC (4/71)
VE07-96-BAC (5/92)
K08-107-BAC (3/59)
Acidimicrobiales
Nitriliruptor alkaliphilus (EF422408)
Nitriliruptorales
Rubrobacter sp. VF70612 S1 (EU512991)
89
Solirubrobacter sp. Gsoil 921 (AB245336)
57
Thermoleophilum album (NR_025543)
88
Clone 5C231714, aquatic bacterial communities (EU804030)
100
A07-25-BAC (1/88)
100
VE07-50-BAC (1/92)
Coriobacterium sp. CCUG 33917 (AJ131149)
Thermotoga maritima (NC 000853)
0.02

Fig. S7A.

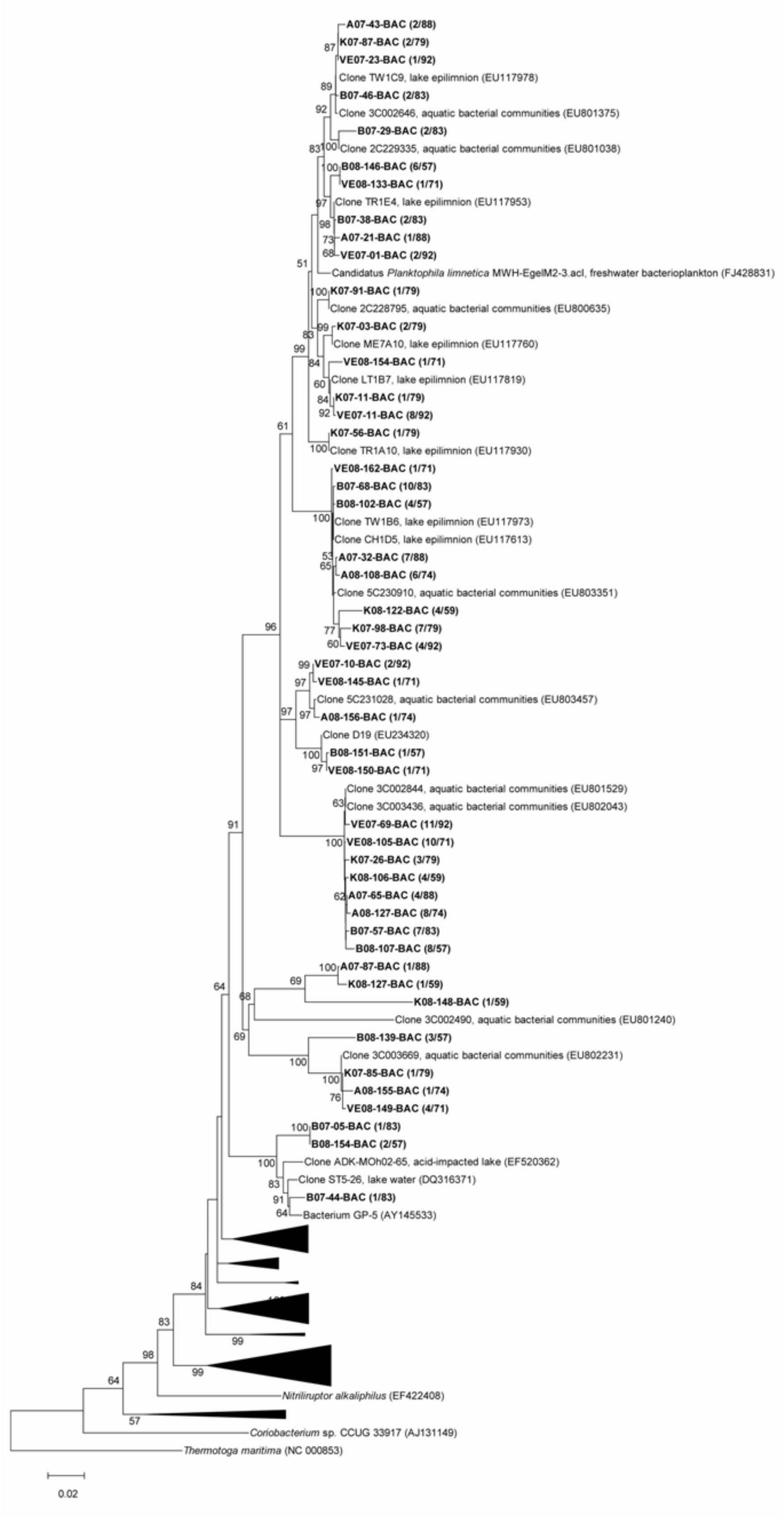

Fig. S7B.

Fig. S7. Phylogenetic tree of the PCR-amplified 16S rRNA gene phylotypes (in bold) (ca. 1390 bp) of Actinobacteria in the water column of Marathonas Reservoir, Greece, based on the neighbour-joining method as determined by distance using Kimura's two-parameter correction. Numbers of identical (≥98% sequence similarity) phylotypes of the total number phylotypes found in each station are shown in parentheses. One thousand bootstrap analyses (distance) were conducted, and percentages ≥50% are indicated at nodes. Numbers in brackets are GenBank accession numbers. Scale bar represents 2% estimated distance.

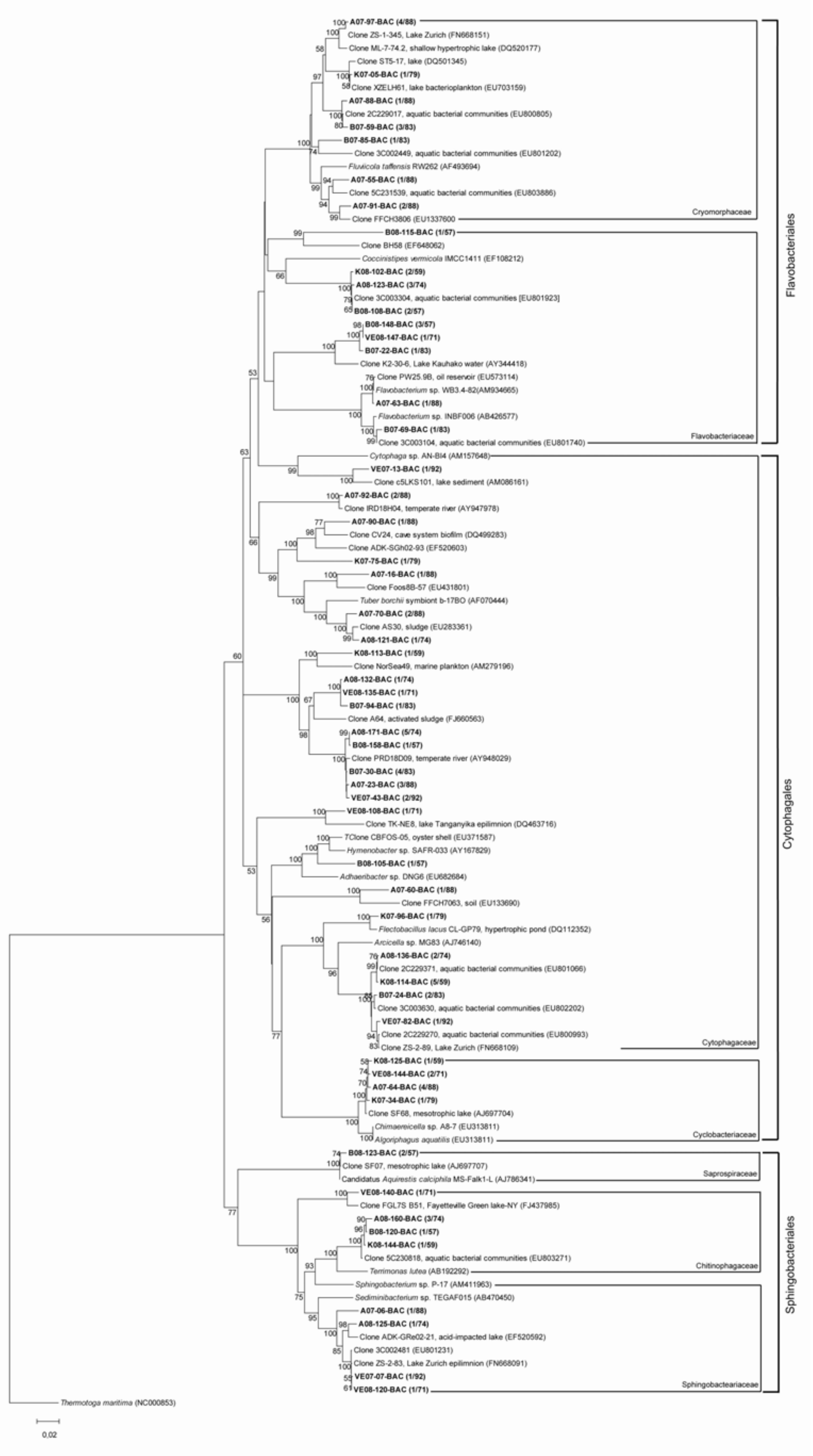

Fig. S8. Phylogenetic tree of the PCR-amplified 16S rRNA gene phylotypes (in bold) (ca. 1390 bp) of Bacteroidetes in the water column of Marathonas Reservoir, Greece, based on the neighbour-joining method as determined by distance using Kimura's two-parameter correction. Numbers of identical (≥98% sequence similarity) phylotypes of the total number phylotypes found in each station are shown in parentheses. One thousand bootstrap analyses (distance) were conducted, and percentages ≥50% are indicated at nodes. Numbers in brackets are GenBank accession numbers. Scale bar represents 2% estimated distance.

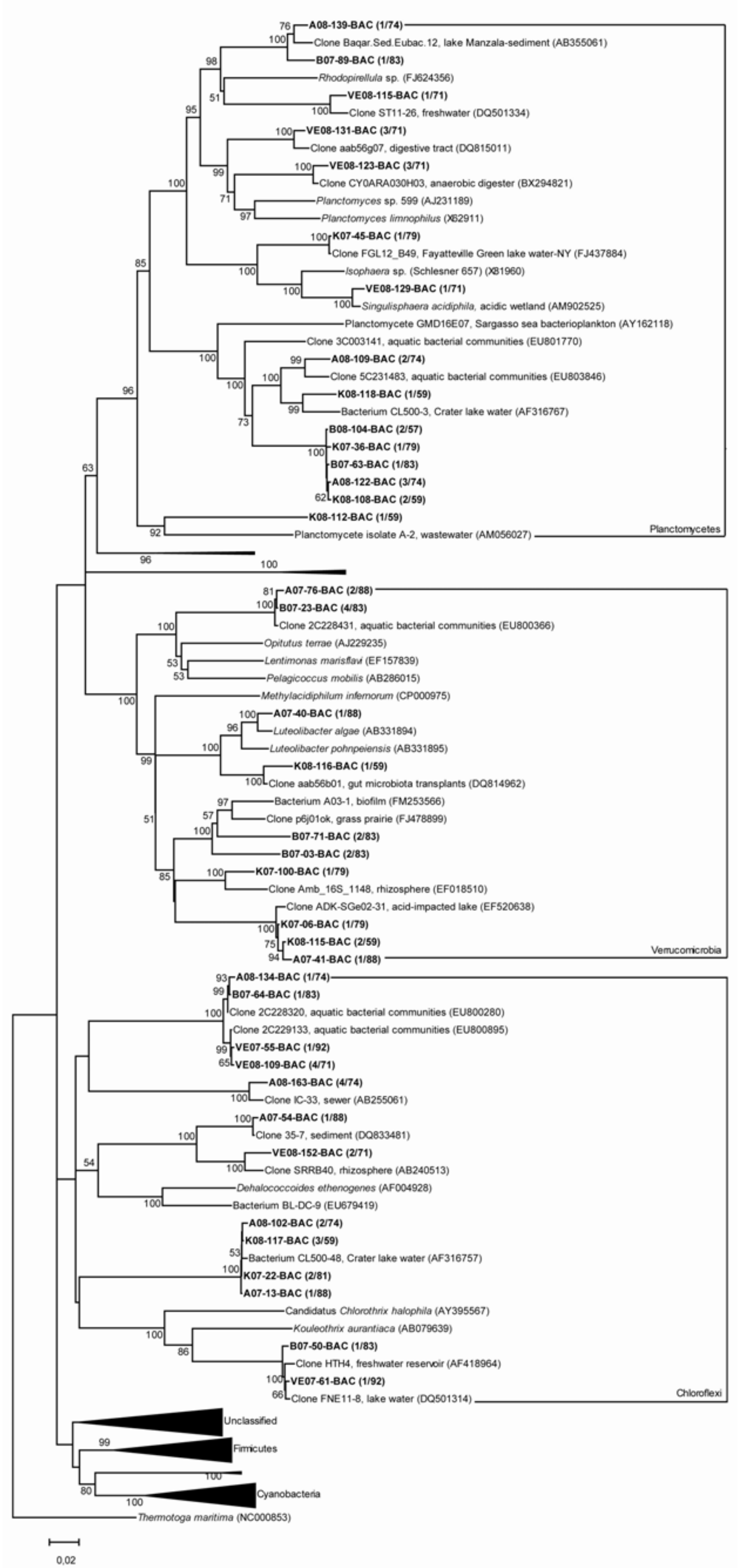

Fig. S9. Phylogenetic tree of the PCR-amplified 16S rRNA gene phylotypes (in bold) (ca. 1390 bp) of Planctomycetes, Verrucomicrobia and Chloroflexi in the water column of Marathonas Reservoir, Greece, based on the neighbour-joining method as determined by distance using Kimura's two-parameter correction. Numbers of identical (≥98% sequence similarity) phylotypes of the total number phylotypes found in each station are shown in parentheses. One thousand bootstrap analyses (distance) were conducted, and percentages ≥50% are indicated at nodes. Numbers in brackets are GenBank accession numbers. Scale bar represents 2% estimated distance.

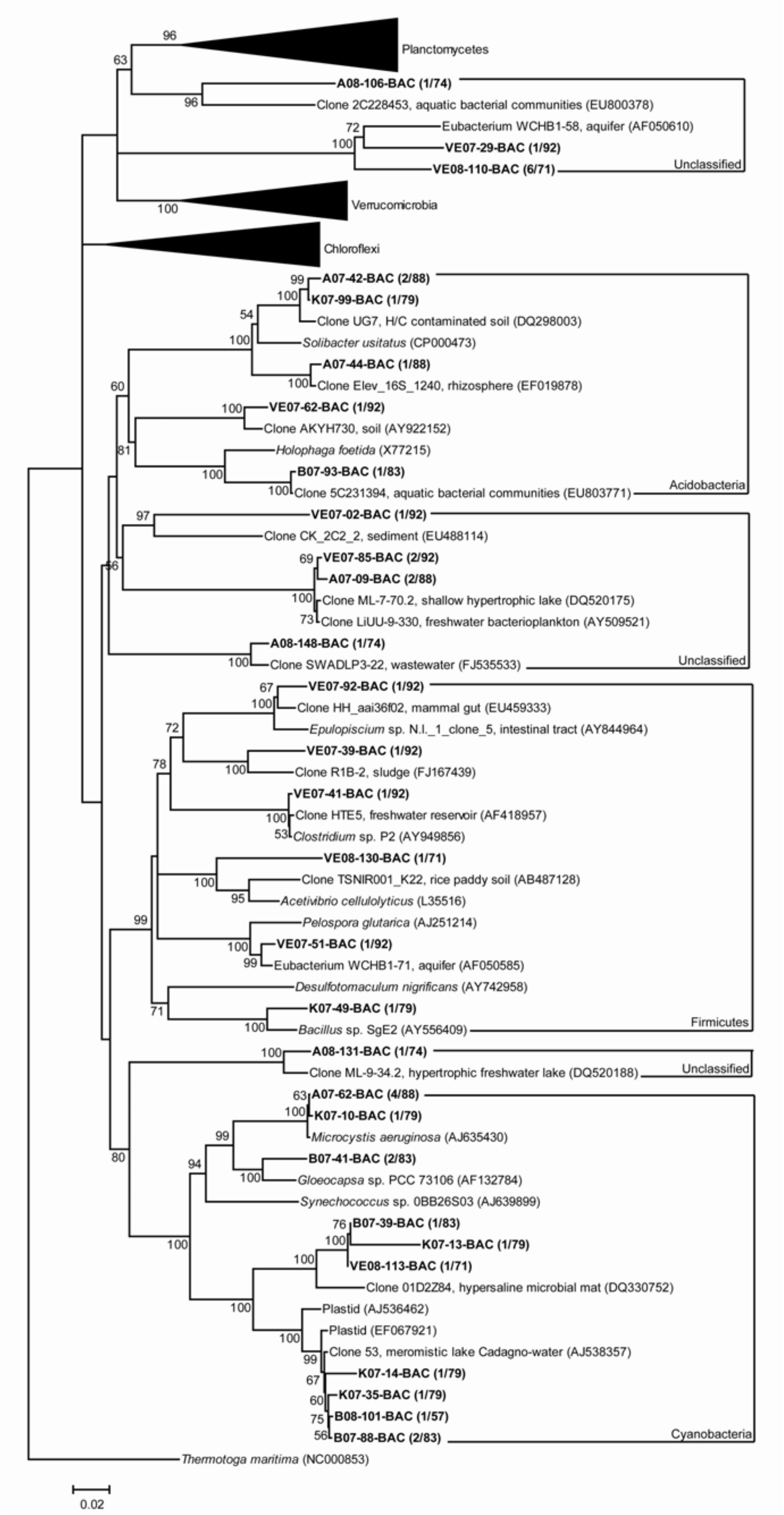

Fig. S10. Phylogenetic tree of the PCR-amplified 16S rRNA gene phylotypes (in bold) (ca. 1390 bp) of Acidobacteria, Firmicutes and Cyanobacteria in the water column of Marathonas Reservoir, Greece, based on the neighbour-joining method as determined by distance using Kimura's two-parameter correction. Numbers of identical (≥98% sequence similarity) phylotypes of the total number phylotypes found in each station are shown in parentheses. One thousand bootstrap analyses (distance) were conducted, and percentages ≥50% are indicated at nodes. Numbers in brackets are GenBank accession numbers. Scale bar represents 2% estimated distance.
